# Limits of Schema Mappings

**DOI:** 10.1007/s00224-017-9812-7

**Published:** 2017-10-02

**Authors:** Phokion G. Kolaitis, Reinhard Pichler, Emanuel Sallinger, Vadim Savenkov

**Affiliations:** 1 UC Santa Cruz and IBM Research-Almaden, Santa Cruz, CA USA; 2 TU Wien, Wien, Austria; 3 University of Oxford, Oxford, UK; 4 Vienna University of Economics and Business, Wien, Austria

**Keywords:** Schema mappings, Limits, Pointwise convergence, Uniform convergence

## Abstract

Schema mappings have been extensively studied in the context of data exchange and data integration, where they have turned out to be the right level of abstraction for formalizing data inter-operability tasks. Up to now and for the most part, schema mappings have been studied as static objects, in the sense that each time the focus has been on a single schema mapping of interest or, in the case of composition, on a pair of schema mappings of interest. In this paper, we adopt a dynamic viewpoint and embark on a study of sequences of schema mappings and of the limiting behavior of such sequences. To this effect, we first introduce a natural notion of distance on sets of finite target instances that expresses how “close” two sets of target instances are as regards the certain answers of conjunctive que- ries on these sets. Using this notion of distance, we investigate pointwise limits and uniform limits of sequences of schema mappings, as well as the companion notions of pointwise Cauchy and uniformly Cauchy sequences of schema mappings. We obtain a number of results about the limits of sequences of GAV schema mappings and the limits of sequences of LAV schema mappings that reveal striking differences between these two classes of schema mappings. We also consider the completion of the metric space of sets of target instances and obtain concrete representations of limits of sequences of schema mappings in terms of generalized schema mappings, that is, schema mappings with infinite target instances as solutions to (finite) source instances.

## Introduction

Schema mappings have been extensively studied in the context of data exchange and data integration, where they have turned out to be the right level of abstraction for formalizing data inter-operability tasks (see the surveys [11, 12] and the monograph [1]). Up to now and for the most part, schema mappings have been studied as static objects, in the sense that each time the focus has been on a single schema mapping or on a finite and, typically, small number of schema mappings. In the case of data exchange [6], a single schema mapping is used to specify the relationship between a source schema and a target schema. In the case of operators on schema mappings [3], such as the composition operator [8, 14], a fixed number of schema mappings is used as input (e.g., two schema mappings in the case of composition) and another schema mapping is returned as output. Even the case of schema-mapping evolution [9] entails a finite (but potentially large) number of schema mappings.

In this paper, we adopt a dynamic viewpoint and embark on a systematic investigation of sequences of schema mappings and of the limiting behavior of such sequences. The original motivation came from the earlier work [2, 5, 7, 10, 14] on schema-mapping optimization and the study of various notions of equivalence between schema mappings that, intuitively, stipulate that two schema mappings cannot be distinguished using conjunctive queries (*C* *Q*-equivalence) or conjunctive queries with at most *n* variables (*C* *Q*_*n*_-equivalence), for some fixed *n* ≥ 1. In particular, in [5] and, implicitly, in [14], it was shown that, given an SO-tgd (second-order tuple-generating dependency) *σ* and a positive integer *n*, one can construct a GLAV schema mapping that is *C* *Q*_*n*_-equivalent to *σ*. Informally, this means that a given SO tgd can be “approximated” by GLAV schema mappings up to any fixed level of precision, even though an SO tgd is a formula of second-order logic that may not be logically equivalent to any formula of first-order logic and, in particular, to any GLAV schema mapping. A more dynamic interpretation is that, given an SO-tgd *σ*, one can obtain a sequence of GLAV schema mappings $(\mathcal {M}_{n})_{n\geq 1}$, whose “limit” is *σ*.

### **Summary of Results**

Our contributions are both conceptual and technical. At the conceptual level, we develop a framework for studying sequences of schema mappings by first introducing a natural notion of *distance* on the powerset $\mathcal {P}(\text {Inst}(\mathbf {T}))$ of the set Inst(**T**) of finite instances over a schema **T**. Intuitively, this notion of distance expresses how “close” two sets of finite **T**-instances are as regards the certain answers of conjunctive queries on these sets. The pair $(\mathcal {P}(\text {Inst}(\mathbf {T})),dist)$ is a pseudometric space, which means that the distance function *d* *i* *s* *t*(⋅,⋅) is symmetric and obeys the triangle inequality, but different sets of finite target instances may have distance zero; however, two such sets have distance zero if and only if they are *C* *Q*-equivalent, i.e., every conjunctive query has the same certain answers on these two sets. Thus, we will also work with the metric space obtained by considering the *C* *Q*-equivalence classes of members of $\mathcal {P}(\text {Inst}(\mathbf {T}))$, and will use the same notation for it.

Sequences of functions from some set to a metric space occupy a central place in the study of metric spaces (see, e.g., [18]). In particular, there are natural notions of a *pointwise limit* and of a *uniform limit* of a sequence (*f*_*n*_)_*n* ≥ 1_ of functions from some set to a metric space; moreover, there are companion notions of a *pointwise Cauchy* and of a *uniformly Cauchy* sequence of such functions. We now describe briefly how these notions can be applied to sequences of schema mappings. In its most general formulation, a schema mapping $\mathcal {M}$ over a source schema **S** and a target schema **T** is a set of pairs (*I*, *J*), where *I* is a finite **S**-instance and *J* is a finite **T**-instance. It follows that a schema mapping $\mathcal {M}$ can be also be viewed as a function *f* from the set Inst(**S**) of all finite **S**-instances to the powerset $\mathcal {P}(\text {Inst}(\mathbf {T}))$ of the set of all finite **T**-instances, where $f(I) =\{J: (I,J) \in \mathcal {M}\}$. This way, a sequence $(\mathcal {M}_{n})_{n\geq 1}$ of schema mappings over a source schema **S** and a target schema **T** can be viewed as a sequence of functions from Inst(**S**) to the (pseudo)metric space $(\mathcal {P}(\text {Inst}(\mathbf {T})),dist)$.

After the conceptual framework has been laid out, we study in depth the limiting behavior of sequences of GAV mappings and the convergence of sequences of LAV mappings. We establish a number of technical results that reveal rather dramatic and perhaps unanticipated differences between GAV schema mappings and LAV schema mappings.

For sequences of GAV mappings, we point out that every uniformly Cauchy sequence of GAV mappings is eventually constant, hence it has a GAV mapping as uniform limit. We also show that every pointwise Cauchy sequence of GAV mappings has a pointwise limit, but it need not have a uniform limit; moreover, there are pointwise Cauchy sequences of GAV mappings such that no GAV mapping is their pointwise limit. This raises the question as to when a sequence of GAV mappings has a GAV mapping as a pointwise limit. We prove that a sequence of GAV mappings has a GAV mapping as a pointwise limit if and only if it has a pointwise limit that allows for *C* *Q*-rewriting^1^.

For sequences of LAV mappings, we show that the notions of uniform limit and pointwise limit coincide; moreover, the same holds true for the notions of uniformly Cauchy and pointwise Cauchy sequences. However, there are uniformly Cauchy sequences of LAV mappings that have no uniform limit. We also establish that a uniformly Cauchy sequence of LAV mappings has a LAV mapping as a uniform limit if and only if it has a uniform limit that admits universal solutions. The aforementioned results lift to sequences of *premise-bounded* sequences of GLAV mappings, i.e., sequences of GLAV mappings for which there is a *k* ≥ 1 such that, for every mapping in the sequence, the left-hand side of every GLAV constraint has at most *k* source atoms (LAV mappings have *k* = 1).

In terms of techniques, we use systematically the structural characterizations of schema-mapping languages established in [19], thus creating a link with a different line of research.

The metric space $(\mathcal {P}(\text {Inst}(\mathbf {T})),dist)$ is incomplete, i.e., there are Cauchy sequences of elements of $\mathcal {P}(\text {Inst}(\mathbf {T}))$ that have no limit in $\mathcal {P}(\text {Inst}(\mathbf {T}))$. It is well known that every incomplete metric space (*X*, *d*) has a completion, which means that it can be embedded into a complete metric space (*X* ^∗^, *d* ^∗^) so that *X* is a dense subset of *X* ^∗^. Moreover, pointwise (respectively, uniformly) Cauchy sequences of functions on *X* have pointwise (respectively, uniform) limits that take values in *X* ^∗^. The construction of *X* ^∗^ from *X* involves equivalence classes of Cauchy sequences of elements of *X*, thus, in general, the members of *X* ^∗^ do not have a concrete representation. In the last part of the paper, we show that the members of $\mathcal {P}(\text {Inst}(\mathbf {T}))^{*}$ can be represented by suitably constructed infinite **T**-instances. As a consequence of this, the pointwise (respectively, uniform) limits of Cauchy sequences of schema mappings can be represented by *generalized* schema mappings, i.e., schema mappings that allow for infinite target instances as solutions to finite source instances.

## Preliminaries

This section contains a minimum amount of necessary background material.

### **Schemas, Instances, and Conjunctive Queries**

A *schema* **R** is a finite sequence 〈*R*_1_,…, *R*_*k*_〉 of relation symbols, where each *R*_*i*_ has a fixed arity. An *instance* *I* over **R**, or an **R** *-instance*, is a sequence (*R*1*I*,…, *R* *k* *I*), where each ${R^{I}_{i}}$ is a finite relation of the same arity as *R*_*i*_. We will often use *R*_*i*_ to denote both the relation symbol and the relation ${R^{I}_{i}}$ that interprets it. The *active domain* *a* *d* *o* *m*(*I*) of an instance *I* is the set of all values occurring in the relations of *I*. A *fact* of an instance *I* (over **R**) is an expression ${R_{i}^{I}}(t_{1}, \ldots , t_{m})$ (or simply *R*_*i*_(*t*_1_,…, *t*_*m*_)), where *R*_*i*_ is a relation symbol of **R** and $(t_{1},\ldots ,t_{m}) \in {R_{i}^{I}}$.

A *conjunctive query* is a first-order formula of the form ∃**z** *𝜃*(**x**, **z**), where *𝜃*(**x**, **z**) is a conjunction of atomic formulas *R*_*i*_(*v*_1_,..., *v*_*m*_) and each *v*_*j*_ is one of the variables in **x** and **z**. A *boolean conjunctive query* is a conjunctive query with no free variables. We write *C* *Q* for the class of all conjunctive queries over some schema. For every *n* ≥ 1, we let *C* *Q*_*n*_ denote the class of all conjunctive queries with at most *n* variables. We also let *C* *Q*_0_ denote the singleton consisting of a trivially true query. If *I* is an instance and *q* is a conjunctive query, then we write *q*(*I*) for the result of evaluating *q* on *I*; in particular, for boolean conjunctive queries *q* we have that *q*(*I*) = *t* *r* *u* *e* if and only if *I* satisfies *q*.

### **Schema Mappings, Universal Solutions, Certain Answers**

Motivated by the terminology in data exchange [6], we typically work with two schemas, a *source schema* **S** and a *target schema* **T** with no relation symbols in common. We refer to **S**-instances as *source instances*, and to **T**-instances as *target instances*. We assume that the values occurring in the active domains of instances come from two fixed countably infinite disjoint sets, the set Const of all *constants* and the set Null of *(labeled) nulls*. We also assume that the active domains of source instances consist entirely of constants; the active domains of target instances may contain both constants and nulls.

In its most general form, a *schema mapping* $\mathcal {M}$ between a source schema **S** and a target schema **T** is a set of pairs (*I*, *J*), where *I* is source instance and *J* a target instance. To avoid anomalies that arise from such a relaxed notion, we will assume that a schema mapping $\mathcal {M}$ must also possess a mild closure property, namely, that $\mathcal {M}$ is closed under isomorphisms that rename nulls by other nulls. This is a natural “genericity” condition that is akin to the condition that database queries are closed under arbitrary isomorphisms. The precise definitions are as follows.

### **Definition 1**

Let **S** be a source schema and **T** a target schema.

- An isomorphism that renames nulls between two target instances J and *J* ^′^ is a one-to-one and onto function *h* : *a* *d* *o* *m*(*J*) → *a* *d* *o* *m*(*J* ^′^) such that:
  (i) If c is a constant in *a* *d* *o* *m*(*J*), then *h*(*c*) = *c*.
  (ii) If w is a null in *a* *d* *o* *m*(*J*), then *h*(*w*) is also a null.
  (iii) For every relation symbol R of **T** of arity m and for every tuple (*a*_1_,…, *a*_*m*_) of constants and nulls, we have that *R* ^*J*^(*a*_1_,…, *a*_*m*_) is a fact of *J* if and only if $R^{J^{\prime }}(h(a_{1}),\ldots ,h(a_{m}))$ is a fact of *J* ^′^.
    In this case, we write *J* ^′^ = *h*(*J*) and say that *J* ^′^ is an *isomorphic copy of* *J* *via an isomorphism that renames nulls*.
- A schema mapping $\mathcal {M}$ between **S** and **T** is a set of pairs (*I*, *J*), where I is source instance and *J* a target instance, such that the following holds: if a pair (*I*, *J*) is in $\mathcal {M}$ and if *J* ^′^ = *h*(*J*) is an isomorphic copy of *J* via an isomorphism h that renames nulls, then also (*I*, *J* ^′^) is in $\mathcal {M}$.

A schema mapping is often (but not always) given as a triple ${\mathcal {M}} = (\mathbf {S}, \mathbf {T}, {\Sigma })$, where Σ is a set of formulas in some logical formalism such that $(I,J) \in \mathcal {M}$ if and only if *I* ∪ *J*⊧Σ. Clearly, if Σ is a set of first-order formulas or a set of second-order formulas, then $\mathcal {M}$ is indeed closed under isomorphisms that rename nulls holds.

Let $\mathcal {M}$ be a fixed schema mapping. In data exchange, the main problem is, given a source instance *I*, to find a *solution* for *I* w.r.t. $\mathcal {M}$, that is, a target instance *J* such that $(I,J)\in \mathcal {M}$ (or determine that no solution exists). We use the notation $\text {Sol}(I,\mathcal {M}) = \{J \mid (I,J) \in \mathcal {M}\}$ to denote the set of all solutions for *I* w.r.t. $\mathcal {M}$. In data integration, the main problem is to compute the *certain answers* of queries [12]. Specifically, given a query *q* over the target schema and a source instance *I*, the *certain answers* of *q* on *I* w.r.t. $\mathcal {M}$ is the set

$$cert(q,I,\mathcal{M}) = \bigcap \{q(J)\mid J \in \text{Sol}(I,\mathcal{M}) \}.$$

If *q* is a boolean conjunctive query, then $cert(q,I,\mathcal {M}) = {\mathit {true}}$, if *q*(*J*) = *t* *r* *u* *e*, for every solution *J* for *I* w.r.t. $\mathcal {M}$; otherwise, $cert(q,I,\mathcal {M}) = {\mathit {false}}$. Note also if *q* is a non-boolean conjunctive query, then either $cert(q,I,\mathcal {M}) = \emptyset $ or every tuple $\textbf {t} \in cert(q,I,\mathcal {M})$ is null-free, that is, it consists entirely of constants. This is a consequence of the closure of $\mathcal {M}$ under isomorphisms that rename nulls. Indeed, assume that $\textbf {t} \in cert(q,I,\mathcal {M})$. Let *J* be a solution for *I* w.r.t. $\mathcal {M}$ and let *J* ^′^ = *h*(*J*) be a target instance that is an isomorphic copy of *J* via an isomorphism *h* that renames nulls from the active domain of *J* to nulls outside the active domain of *J* (such a target instance *J* ^′^ and such an isomorphism *h* exist because *J* is a finite set of facts, hence its active domain is a finite set). By the closure property of $\mathcal {M}$, the target instance *J* ^′^ is also a solution for *I* w.r.t. $\mathcal {M}$, hence **t** ∈ *q*(*J* ^′^). It follows that **t** must consist of values in the intersection *a* *d* *o* *m*(*J*) ∩ *a* *d* *o* *m*(*J* ^′^) of the active domains of *J* and *J* ^′^, hence **t** must consist entirely of constants. Note that the only property of conjunctive queries used in this argument is that they are *safe*, that is, they return tuples from the active domain of the instance on which they are evaluated.

On the face of it, the definition of certain answers may entail computing an intersection of infinitely many sets. One of the main findings in [6] is that there is a notion of a “good” solution in data exchange, called *universal solution*, that can also be used to compute the certain answers of conjunctive queries in a much more direct way.

Let *J*_1_ and *J*_2_ be two target instances. A function *h* is a *homomorphism* from *J*_1_ to *J*_2_ if the following hold: (i) for every constant *c*, we have that *h*(*c*) = *c*; and (ii) for every relation symbol *R* in **R** and every tuple $(a_{1},\ldots ,a_{n})\in R^{J_{1}}$, we have that $(h(a_{1}),\ldots ,h(a_{n}))\in R^{J_{2}}$. We write *J*_1_ → *J*_2_ to denote that there is a homomorphism from *J*_1_ to *J*_2_. We say that *J*_1_ is *homomorphically equivalent* to *J*_2_, written *J*_1_ ⇔ *J*_2_, if *J*_1_ → *J*_2_ and *J*_2_ → *J*_1_.

Let *I* be a source instance. A *universal* solution for *I* w.r.t. $\mathcal {M}$ is a solution *J* such that for every solution $J^{\prime } \in \text {Sol}(I,\mathcal {M})$, we have that *J* → *J* ^′^. Intuitively, a universal solution for *I* is a “most general” solution for *I*. We write $\text {UnivSol}(I,\mathcal {M})$ to denote the set of all universal solutions for *I* w.r.t. $\mathcal {M}$ (note that universal solutions need not always exist, so it is possible that $\text {UnivSol}(I,\mathcal {M})= \emptyset $). The following useful property of universal solutions was first identified in [6].

### **Proposition 1**

*Assume that* $\mathcal {M}$ *is a schema mapping,* I *is a source instance,* *and* *J* *is a universal solution for* I *w.r.t.* $\mathcal {M}$.*If* q *is a conjunctive query, then* $cert(q,I,\mathcal {M}) = q(J)_{\downarrow }$ *,* *where* *q*(*J*)_*↓*_ *is the set of all null-free tuples in* *q*(*J*).

### *Proof 1*

First, assume that $\textbf {t} \in cert(q,I,\mathcal {M})$. Then, as discussed earlier, **t** must be a null-free tuple. Since *J* is a solution for I w.r.t. $\mathcal {M}$, we have that **t** ∈ *q*(*J*), hence we have that **t** ∈ *q*(*J*) *↓*. Next, assume that **t** is a null-free tuple in *q*(*J*). If *J* ^′^ is an arbitrary solution for I w.r.t. $\mathcal {M}$, then, since *J* is a universal solution for I w.r.t. I, there is a homomorphism h from *J* to *J* ^′^. Since conjunctive queries are preserved under homomorphisms, it follows that *h*(**t**) = **t** ∈ *q*(*J* ^′^). Thus, $\textbf {t} \in cert(q,I,\mathcal {M})$. □

### **Structural Properties of Schema Mappings**

We now present a number of structural properties that a schema mapping may or may not possess. These properties were investigated in their own right in [19], where they were used to obtain characterizations of schema-mapping languages that will be of great interest to us in this paper.

Let $\mathcal {M}$ be a schema mapping.

- $\mathcal {M}$ *allows for* *C* *Q*-rewriting if for every target conjunctive query *q*, there exists a union *q* ^′^ of source conjunctive queries such that $cert(I,\mathcal {M},q) = q^{\prime }(I)$, for every source instance *I*.
- $\mathcal {M}$ *admits universal solutions* if for every source instance *I*, there is a universal solution for *I* w.r.t. $\mathcal {M}$.
- $\mathcal {M}$ is *closed under target homomorphisms* if $(I,J) \in \mathcal {M}$ and *J* → *J* ^′^ imply that $(I,J^{\prime }) \in \mathcal {M}$.
- $\mathcal {M}$ is *closed under unions* if $(I_{1},J_{1}) \in \mathcal {M}$ and $(I_{2},J_{2}) \in \mathcal {M}$ imply that $(I_{1}\cup I_{2}, J_{1} \cup J_{2}) \in \mathcal {M}$.
- $\mathcal {M}$ is *closed under target intersections* if $J_{1} \in \text {Sol}(I,\mathcal {M})$ and $J_{2} \in \text {Sol}(I,\mathcal {M})$ imply that $(J_{1} \cap J_{2}) \in \text {Sol}(I,\mathcal {M})$.
- $\mathcal {M}$ is *n-modular* if whenever $(I,J) \notin \mathcal {M}$, there is a subinstance *I* ^′^⊆ *I* with at most *n* elements in its active domain such that $(I^{\prime },J) \notin \mathcal {M}$ (“small counterexample”).

### **Schema Mapping Languages**

A GLAV *(global-and-local-as-view)* constraint is a first-order formula of the form ∀**x**(*φ*(**x**) →∃**y** *ψ*(**x**, **y**)), where *φ*(**x**) is a conjunction of atoms over the source schema **S**, each variable in **x** occurs in at least one atom in *φ*(**x**), and *ψ*(**x**, **y**) is a conjunction of atoms over the target schema **T** with variables in **x** and **y**. We refer to *φ*(**x**) as the *left-hand side*, or *premise*, and ∃**y** *ψ*(**x**, **y**) as the *right-hand side*, or *conclusion* of the constraint. Another name for GLAV constraints is *source-to-target tuple-generating dependencies* or, in short, *s-t tgds*.

A LAV (*local-as-view*) constraint is a GLAV constraint whose left-hand side is a single atom over the source, while a GAV (*global-as-view*) constraint is a GLAV constraint whose right-hand side contains no existential quantifiers and consists of a single atom over the target. For example, ∀*x*, *y*(*E*(*x*, *y*) →∃*z*(*F*(*x*, *z*) ∧ *F*(*z*, *y*))) is a LAV constraint, and ∀*x*, *y*, *z*(*E*(*x*, *z*) ∧ *E*(*z*, *y*) → *F*(*x*, *y*)) is a GAV constraint.

A GLAV (*global-and-local-as-view*) mapping is a schema mapping $\mathcal {M}=(\mathbf {S}, \mathbf {T}, {\Sigma })$ such that Σ is a finite set of GLAV constraints. The notions of a *LAV mapping* and of a *GAV mapping* are defined analogously.

Every GLAV mapping $\mathcal {M}$ admits universal solutions [6]; furthermore, given a source instance *I*, a *canonical universal solution* $chase(I,\mathcal {M})$ can be produced via the *oblivious chase procedure* as follows: whenever the antecedent of an s-t tgd in $\mathcal {M}$ becomes true, fresh null values are introduced and facts involving these nulls are added to $chase(I,\mathcal {M})$, so that the conclusion of the s-t tgd becomes true. Every GLAV mapping is also known to allow for *C* *Q*-rewriting and to be *n*-modular, for some *n* ≥ 1. Moreover, every LAV mapping is closed under unions, while every GAV mapping is closed under target intersections.

*Second-Order tgds*, or *SO tgds*, were introduced in [8] and were shown to be exactly the constraints needed to express the composition of a finite number of GLAV mappings. Instead of giving the precise definition of an SO tgd, we illustrate this notion with an example from [8]. The formula

$$\exists f(\forall e (\mathit{Emp}(e) \rightarrow \mathit{Mgr}(e,f(e))) \land \forall e (\mathit{Emp}(e) \land (e=f(e)) \rightarrow \mathit{SelfMgr}(e)) )$$

expresses the property that every employee has a manager, and if an employee is the manager of himself/herself, then this employee is a self-manager. Clearly, SO tgds are existential second-order formulas with existentially quantified function symbols, which can be thought of as acting like Skolem functions. The use of these function symbols, however, is limited by the syntax of SO tgds: they can only appear in equations between terms in the antecedent of an implication or as arguments of atoms in the conclusion of an implication. As regards expressive power, SO tgds are, in general, strictly more expressive than GLAV constraints, but less expressive than arbitrary existential second-order formulas. In particular, the above formula is an SO tgd that is not logically equivalent to any (finite or infinite) set of GLAV constraints [8].

Every SO tgd allows for *C* *Q*-rewriting and admits universal solutions; however, an SO tgd may not be closed under target homomorphisms and there may not exist any *n* ≥ 1 such that the SO tgd is *n*-modular (see [8, 19]).

### **Pseudometric Spaces and Metric Spaces**

A *pseudometric space* is a pair (*X*, *d*), where *X* is a set and *d* is a function from *X* × *X* to the set *R* ^+^ of non-negative real numbers with the following properties: (i) *d*(*x*, *x*) = 0, for every *x* in *X*; (ii) *d*(*x*, *y*) = *d*(*y*, *x*), for every *x* and *y* in *X*; (iii) *d*(*x*, *y*) ≤ *d*(*x*, *z*) + *d*(*y*, *z*), for every *x*, *y*, *z* in *X* (triangle inequality). A *metric space* is a pseudometric space (*X*, *d*) such that if *d*(*x*, *y*) = 0, then *x* = *y*. It is easy to see that if (*X*, *d*) is a pseudometric space, then the relation *R*_*d*_ = {(*x*, *y*) ∈ *X* × *X*∣*d*(*x*, *y*) = 0} is an equivalence relation on *X*. From this, it follows that every pseudometric space (*X*, *d*) gives rise to a metric space $(\widehat {X},\widehat {d})$, where $\widehat {X}$ is the set of equivalence classes of elements of X modulo the equivalence relation *R*_*d*_ and $\widehat {d}([x],[y]) = d(x,y)$.

Let (*X*, *d*) be a pseudometric space. A sequence of elements *x*_1_, *x*_2_,… of *X* *converges* to an element *x* of *X*, denoted by $\lim \limits _{n\to \infty } x_{n} = x$, if for every *𝜖* > 0, there is an integer *n*_0_ such that *d*(*x*_*n*_, *x*) < *𝜖*, for every *n* ≥ *n*_0_. We say that *x* is a *limit* of this sequence. The limit is unique if (*X*, *d*) is a metric space. A sequence *x*_1_, *x*_2_,… of elements of *X* is *Cauchy* if for every *𝜖* > 0, there is an integer *n*_0_ such that $d(x_{n},x_{n^{\prime }}) < \epsilon $, for every *n*, *n* ^′^≥ *n*_0_.

Using the triangle inequality, it is easy to see that if a sequence of elements in a (pseudo)metric space has a limit, then the sequence is Cauchy. The converse, however, does not hold for arbitrary (pseudo)metric spaces. A (pseudo)metric space (*X*, *d*) is *complete* if every Cauchy sequence of elements of *X* has a limit in *X*; otherwise, it is *incomplete*.

It is well known that every incomplete (pseudo)metric space (*X*, *d*) can be embedded into a complete (pseudo)metric space (*X* ^∗^, *d* ^∗^), called the *completion* of (*X*, *d*), in such a way that *X* is a *dense* subset of *X* ^∗^, i.e., every member of *X* ^∗^ is the limit of a sequence of members of *X*. The members of *X* ^∗^ are equivalence classes of Cauchy sequences of *X*, where two Cauchy sequences *x*_1_, *x*_2_,... and *y*_1_, *y*_2_,… of elements of *X* are *equivalent* if $\lim \limits _{n\to \infty } d(x_{n},y_{n}) = 0$, while the distance function *d* ^∗^ is defined as $d^{*}([x_{1},x_{2},\ldots ],[y_{1},y_{2},\ldots ]) = \lim \limits _{n\to \infty } d(x_{n},y_{n})$. The proof of correctness of this construction can be found in [18] or any other book on metric spaces.

As a concrete example, the metric space of the real numbers is the completion of the metric space of the rational numbers (both with the standard distance).

## Metric Space of Target Instances

To study the limits of sequences of schema mappings, we first introduce a pseudometric space of sets of target instances. By considering schema mappings as functions that map each source instance to the set of its solutions, we can view sequences of schema mappings as *sequences of functions*. The (pointwise or uniform) limit of a sequence of schema mappings is then simply defined in the standard way as the limit of a sequence of functions taking values in a pseudometric space. Moreover, by passing to the associated metric space of equivalence classes of sets of target instances, we ensure the uniqueness of the limit. If **T** is a schema, we write Inst(**T**) for the set of all finite instances of **T**. We also write $\mathcal {P}(\text {Inst}(\mathbf {T}))$ for the power set of Inst(**T**). The notion of distance on $\mathcal {P}(\text {Inst}(\mathbf {T}))$ that we are about to introduce is heavily based on the notion of the certain answers to conjunctive queries and on the idea that two members $\mathcal {J}$ and $\mathcal {J}^{\prime }$ of $\mathcal {P}(\text {Inst}(\mathbf {T}))$ are “close” to each other if only “big” conjunctive queries can yield different certain answers on $\mathcal {J}$ and $\mathcal {J}^{\prime }$.

### **Definition 2**

Let **T** be a schema.

- Let q be a query over **T** and let $\mathcal {J}$ be a member of $\mathcal {P}(\text {Inst}(\mathbf {T}))$. The *certain* *answers* of q over $\mathcal {J}$ are defined as
  $$cert(q,\mathcal{J}) = \bigcap \{q(J) \mid J \in \mathcal{J}\}.$$
- We say that two sets of instances $\mathcal {J}$ and $\mathcal {J}^{\prime }$ in $\mathcal {P}(\text {Inst}(\mathbf {T}))$ are *C* *Q*-equivalent, denoted $\mathcal {J} \equiv _{\mathsf {CQ}} \mathcal {J}^{\prime }$, if $cert(q,\mathcal {J}) = cert(q,\mathcal {J}^{\prime })$ holds for all conjunctive queries *q*.
- We say that $\mathcal {J}$ and $\mathcal {J}^{\prime }$ are *C* *Q*_*n*_-equivalent, denoted $\mathcal {J} \equiv _{\mathsf {CQ}_{n}} \mathcal {J}^{\prime }$, if it holds that $cert(q,\mathcal {J}) = cert(q,{\mathcal {J}^{\prime }})$ for all conjunctive queries *q* with at most *n* variables (i.e., for all *q* in *C* *Q*_*n*_.)

### **Definition 3**

Let $\mathcal {J}$ and $\mathcal {J}^{\prime }$ be two sets of instances in $\mathcal {P}(\text {Inst}(\mathbf {T}))$. The similarity $sim(\mathcal {J},\mathcal {J}^{\prime })$ and the distance $dist(\mathcal {J},\mathcal {J}^{\prime })$ between $\mathcal {J}$ and $\mathcal {J}^{\prime }$ are defined as follows:

- $sim(\mathcal {J},\mathcal {J}^{\prime }) = \max \{k \mid \mathcal {J} \equiv _{\mathsf {CQ}_{k}} \mathcal {J}^{\prime }\}$;
- $dist(\mathcal {J},\mathcal {J}^{\prime }) = 2^{-sim(\mathcal {J},\mathcal {J}^{\prime })}$.

It is easy to verify that the pair $(\mathcal {P}(\text {Inst}(\mathbf {T})),dist)$ is a pseudometric space; in fact, *dist* is an *ultrametric* distance function, that is,

$$dist(\mathcal{J},\mathcal{J}^{\prime}) \leq \max\{dist(\mathcal{J},\mathcal{J}^{\prime\prime}), dist(\mathcal{J}^{\prime\prime},\mathcal{J}^{\prime})\}$$

holds for all $\mathcal {J}$, $\mathcal {J}^{\prime }$, $\mathcal {J}^{\prime \prime }$ in $\mathcal {P}(\text {Inst}(\mathbf {T}))$. Moreover, $dist(\mathcal {J},\mathcal {J}^{\prime }) = 0$ if and only if $\mathcal {J}$ and $\mathcal {J}^{\prime }$ are *C* *Q*-equivalent.

### **Definition 4**

Let **T** be a schema. If *J* is a **T**-instance, then we write *v*(*J*) to denote the member of $\mathcal {P}(\text {Inst}(\mathbf {T}))$ consisting of all isomorphic copies of *J* via isomorphisms that rename nulls. In other words, *v*(*J*) consists of all **T**-instances *J* ^′^ such that *J* ^′^ is isomorphic to *J* via an isomorphism h that maps each constant to itself and maps each null to a (possibly different) null.

The next lemma will be used repeatedly in the sequel.

### **Lemma 1**

*Let* **T** *be a schema.*

1. *If* *J* *is a* **T** *-instance* *whose active domain consists entirely of nulls* *and* q *is a non-boolean conjunctive query, then* *c* *e* *r* *t*(*q*, *v*(*J*)) = *∅*.
2. *If* *J* *is a* **T** *-instance* *whose active domain consists entirely of nulls* *and* q *is a boolean conjunctive query, then* *c* *e* *r* *t*(*q*, *v*(*J*)) = *q*(*J*).
3. *If* *J* *and* *J* ^′^ *are* **T** *-instances* *whose active domains consist entirely of nulls, then, for every* *k* ≥ 1*,* *the following statements are equivalent:*
  - $v(J) \equiv _{\mathsf {CQ}_{k}} v(J^{\prime })$.
  - *J and* *J* ^′^ *satisfy the same boolean conjunctive queries in* *C* *Q*_*k*_.

### *Proof 2*

For the first two parts of the lemma, let *J* be a **T**-instance whose active domain consists entirely of nulls. For every non-boolean query q in *C* *Q*_*k*_, we have that *c* *e* *r* *t*(*q*, *v*(*J*)) = *∅*, because *v*(*J*) contains instances with disjoint active domains. For every boolean query q, we have *c* *e* *r* *t*(*q*, *v*(*J*)) = *q*(*J*) for the following reason: first, *J* is a member of *v*(*J*), so if *c* *e* *r* *t*(*q*, *v*(*J*)) = *t* *r* *u* *e*, then *q*(*J*) = *t* *r* *u* *e* as well; second, since every member of *v*(*J*) is isomorphic to *J* and since boolean conjunctive queries are preserved under isomorphisms, we have that if *q*(*J*) = *t* *r* *u* *e*, then *c* *e* *r* *t*(*q*, *v*(*J*)) = *t* *r* *u* *e*.

For the third part of the lemma, let *J* and *J* ^′^ be **T**-instances whose active domains consist entirely of nulls and let k be a positive integer. If $v(J) \equiv _{\mathsf {CQ}_{k}} v(J^{\prime })$, then *J* and *J* ^′^ must satisfy the same boolean conjunctive queries in *C* *Q*_*k*_ because *J* ∈ *v*(*J*) and *J* ^′^∈ *v*(*J* ^′^). For the converse, assume that *J* and *J* ^′^ satisfy the same boolean conjunctive queries in *C* *Q*_*k*_. We have to show that *c* *e* *r* *t*(*q*, *v*(*J*)) = *c* *e* *r* *t*(*q*, *v*(*J* ^′^)), for every conjunctive query q in *C* *Q*_*k*_. If q is a non-boolean conjunctive query in *C* *Q*_*k*_, then, by the first part of the lemma, we have that *c* *e* *r* *t*(*q*, *v*(*J*)) = *∅* = *c* *e* *r* *t*(*q*, *v*(*J* ^′^)). If q is a boolean query in *C* *Q*_*k*_, then, by the second part of the lemma and the hypothesis about *J* and *J* ^′^, we have that *c* *e* *r* *t*(*q*, *v*(*J*)) = *q*(*J*) = *q*(*J* ^′^) = *c* *e* *r* *t*(*q*, *v*(*J* ^′^)). □

The preceding lemma will be used in the next example, which presents a sequence from $\mathcal {P}(\text {Inst}(\mathbf {T}))$ that has a limit in $\mathcal {P}(\text {Inst}(\mathbf {T}))$.

### *Example 1*

Let **T** be a schema consisting of a single binary relation E and let *C*_*m*_ be the undirected cycle of length m, *m* ≥ 1, where the vertices of the cycle are pairwise distinct labeled nulls. Consider the sequence (*v*(*C*_2*n*+1_))_*n* ≥ 1_ arising from the cycles of odd size. Then, for every *m* ≥ 1, we have that $\lim \limits _{n\to \infty }v(C_{2n+1}) = v(C_{2m})$. In particular, $\lim \limits _{n\to \infty }v(C_{2n+1}) = v(C_{2})$.

We first show that *v*(*C*_2*m*_) ≡_*CQ*_ *v*(*C*_2_), for every *m* ≥ 1. By Lemma 1, it suffices to show that *C*_2*m*_ and *C*_2_ satisfy the same boolean conjunctive queries. This is true because *C*_2*m*_ and *C*_2_ are homomorphically equivalent (and boolean conjunctive queries are preserved under homomorphisms). Indeed, there is a homomorphism from *C*_2_ to *C*_2*m*_ because *C*_2_ is a subgraph of *C*_2*m*_, and there is a homomorphism from *C*_2*m*_ to *C*_2_ because *C*_2*m*_ is 2-colorable.

We will show that $\lim \limits _{n\to \infty }v(C_{2n+1}) = v(C_{2})$ by showing that for every k, there exists *n*_0_ such that for all *n* ≥ *n*_0_, we have that $v(C_{2n+1}) \equiv _{\mathsf {CQ}_{k}} v(C_{2})$. For this, we take *n*_0_ = *k* and show that if *n* ≥ *k*, then $v(C_{2n+1}) \equiv _{\mathsf {CQ}_{k}} v(C_{2})$. By the third part of Lemma 1, it suffices to show if q is a boolean conjunctive query in *C* *Q*_*k*_, then *q*(*C*_2*n*+1_) = *q*(*C*_2_). Since *C*_2_ is a subgraph of *C*_2*n*+1_, we have that if *q*(*C*_2_) = *t* *r* *u* *e*, then also *q*(*C*_2*n*+1_) = *t* *r* *u* *e*. Assume that *q*(*C*_2*n*+1_) = *t* *r* *u* *e*. Since *q* ∈*C* *Q*_*k*_, there is a subgraph H of *C*_2*n*+1_ with at most k distinct nodes such that *q*(*H*) = *t* *r* *u* *e*. Since 2*n* + 1 > *n* ≥ *k*, we have that H is a proper subgraph of *C*_2*n*+1_. Consequently, H is 2-colorable and so there is a homomorphism from H to *C*_2_, which, in turn, implies that *q*(*C*_2_) = *t* *r* *u* *e*.

In contrast to what we have *J*ust seen, there are Cauchy sequences of elements of $\mathcal {P}(\text {Inst}(\mathbf {T}))$ that have no limit in $\mathcal {P}(\text {Inst}(\mathbf {T}))$. Thus, the pseudo-metric space $(\mathcal {P}(\text {Inst}(\mathbf {T})),dist)$ is incomplete.

### **Proposition 2**

*Let* **T** *be a schema consisting of a single binary relation E* *and let* *K*_*n*_ *be the clique of size n*, *for* *n* ≥ 1, *where the vertices are pairwise distinct labeled nulls. The sequence* (*v*(*K*_*n*_))_*n* ≥ 1_ *is Cauchy, but has no limit in* $\mathcal {P}(\text {Inst}(\mathbf {T}))$.

### *Proof 3*

The sequence (*v*(*K*_*n*_))_*n* ≥ 1_ is Cauchy, because if *m* ≥ *n*, then *v*(*K*_*m*_) and *v*(*K*_*n*_) satisfy the same conjunctive queries in *C* *Q*_*n*_. To show this, by the third part of Lemma 1, it suffices to show that if *m* ≥ *n*, then *K*_*m*_ and *K*_*n*_ satisfy the same boolean conjunctive queries in *C* *Q*_*n*_. Let q be a boolean conjunctive query in *C* *Q*_*n*_. Since *K*_*n*_ is a subgraph of *K*_*m*_, if *q*(*K*_*n*_) = *t* *r* *u* *e*, then *q*(*K*_*m*_) = *t* *r* *u* *e*. Conversely, if *q*(*K*_*m*_) = *t* *r* *u* *e*, then there is a subgraph H of *K*_*m*_ with at most n distinct nodes such that *q*(*H*) = *t* *r* *u* *e*. But then H is also a subgraph of *K*_*n*_, hence *q*(*K*_*m*_) = *t* *r* *u* *e*.

It remains to show that the sequence (*v*(*K*_*n*_))_*n* ≥ 1_ has no limit in $\mathcal {P}(\text {Inst}(\mathbf {T}))$. Assume to the contrary that there does exist a set $\mathcal {J}$ of finite instances over **T** such that $\lim \limits _{n\to \infty } v(K_{n})= \mathcal {J}$. We distinguish three cases.

First, if $\mathcal {J}=\emptyset $, then $cert(q,\mathcal {J})= {\mathit {true}}$, for every conjunctive query q. In particular, this holds for the query *q* = ∃*x* *E*(*x*, *x*), which asserts the existence of a self-loop. In contrast, for this conjunctive query, we have that *c* *e* *r* *t*(*q*, *v*(*K*_*n*_)) = *f* *a* *l* *s* *e*, for every *n* ≥ 1, since *K*_*n*_ ∈ *v*(*K*_*n*_) and none of the graphs *K*_*n*_, *n* ≥ 1 contains a self-loop.

Second, if $\mathcal {J} \neq \emptyset $ and if every member *J* of $\mathcal {J}$ contains a self-loop, then we again consider the query *q* = ∃*x* *E*(*x*, *x*). We thus have $cert(q, \mathcal {J}) = {\mathit {true}}$, whereas *c* *e* *r* *t*(*q*, *v*(*K*_*n*_)) = *f* *a* *l* *s* *e*, for every *n* ≥ 1.

It remains to consider the case that $\mathcal {J} \neq \emptyset $ and at least one member $J \in \mathcal {J}$ does not contain a self-loop. Let m be the biggest integer such that *J* contains a clique of size m. We define the query q as

$$q = \exists x_{1}, {\dots} x_{m+1} \bigwedge\limits_{i \neq j} (E(x_{i},x_{j}) \wedge E(x_{j},x_{i})).$$

For graphs without self-loops, *q* asserts the existence of a clique of size *m* + 1. We now have that *q* evaluates to false overy *J*. Hence, $cert(q,\mathcal {J}) = {\mathit {false}}$ holds, while *c* *e* *r* *t*(*q*, *v*(*K*_*n*_)) = *t* *r* *u* *e*, for every *n* ≥ *m* + 1. □

Since (*v*(*K*_*n*_)_*n* ≥ 1_) is a Cauchy sequence, it has a limit in the completion of $(\mathcal {P}(\text {Inst}(\mathbf {T})),dist)$. As we will see in Section 6, a concrete representation of this limit is the set consisting of all disjoint unions of cliques of all finite sizes in which every node is a null.

The following definitions are perfectly meaningful for every pseudometric space (*X*, *d*) and for every sequence of functions taking values in *X*. For concreteness, we give the definitions for sequences of functions taking values in $\mathcal {P}(\text {Inst}(\mathbf {T}))$.

### **Definition 5**

Let A be a set, let (*f*_*n*_)_*n* ≥ 1_ be a sequence of functions from A to $\mathcal {P}(\text {Inst}(\mathbf {T}))$, and let f be a function from *A* to $\mathcal {P}(\text {Inst}(\mathbf {T}))$.

- We say that (*f*_*n*_)_*n* ≥ 1_ converges pointwise to f , denoted as $\lim \limits _{n\to \infty }^{p} f_{n} = f$, if for every element *x* ∈ *A*, we have that $\lim \limits _{n\to \infty } f_{n}(x) = f(x)$.
- We say that (*f*_*n*_)_*n* ≥ 1_ converges uniformly to f , denoted as $\lim \limits _{n\to \infty }^{u} f_{n} = f$, if for every *𝜖* > 0, there exists an integer *n*_0_ ≥ 1 such that for every integer *n* ≥ *n*_0_ and for every element *x* ∈ *A*, we have *d* *i* *s* *t*(*f*_*n*_(*x*), *f*(*x*)) < *𝜖*.
- We say that (*f*_*n*_)_*n* ≥ 1_ is pointwise Cauchy, if for every element *x* ∈ *A*, the sequence (*f*_*n*_(*x*))_*n* ≥ 1_ is Cauchy.
- We say that (*f*_*n*_)_*n* ≥ 1_ is uniformly Cauchy, if for every *𝜖* > 0, there exists an integer *n*_0_ ≥ 1 such that for all integers *n*, *n* ^′^≥ *n*_0_ and for every element *x* ∈ *A*, we have $dist(f_{n}(x), f_{n^{\prime }}(x)) < \epsilon $.

Clearly, if (*f*_*n*_)_*n* ≥ 1_ converges pointwise (resp., uniformly), then (*f*_*n*_)_*n* ≥ 1_ is pointwise (resp., uniformly) Cauchy. The converse is not in general true for arbitrary (pseudo)metric spaces; in particular, it is not true for the pseudometric space $(\mathcal {P}(\text {Inst}(\mathbf {T})),dist)$, as we shall see later on.

We now bring schema mappings into the picture. Every schema mapping $\mathcal {M}$ over a source schema **S** and a target schema **T** can be identified with a function $f \colon \text {Inst}(\mathbf {S}) \longrightarrow \mathcal {P}(\text {Inst}(\mathbf {T}))$, where $f(I) = \text {Sol}(I,\mathcal {M})$ (recall that $\text {Sol}(I,\mathcal {M})$ is the set of all solutions of *I* w.r.t. $\mathcal {M}$, i.e., the set of all finite **T** instances *J* such that $(I,J)\in \mathcal {M}$). Thus, a sequence $(\mathcal {M}_{n})_{n\geq 1}$ of schema mappings over a source schema **S** and target schema **T** can be viewed as a sequence of functions from Inst(**S**) to $\mathcal {P}(\text {Inst}(\mathbf {T}))$. Therefore, we can talk about a sequence of schema mappings being pointwise Cauchy and uniformly Cauchy if the sequence of the associated functions has these properties. Similarly, we say that a sequence of schema mappings has a pointwise limit (resp., a uniform limit) if the sequence of the associated functions converges pointwise (resp., converges uniformly) to a schema mapping.

The preceding notion of convergence of a sequence of schema mappings allows us to draw a connection to earlier work on schema mapping optimization [5, 7]. Here, we are considering *C* *Q*-equivalence and *C* *Q*_*n*_-equivalence of *sets of instances*. In previous works, these notions of equivalence have been mainly applied to schema mappings (see, e.g., [5, 7, 14]). Specifically, two schema mappings $\mathcal {M}, \mathcal {M}^{\prime }$ are *C* *Q*-equivalent (resp., *C* *Q*_*n*_-equivalent) if for every target conjunctive query *q* (resp., every target conjunctive query *q* in *C* *Q*_*n*_) and every source instance *I*, we have that $cert(q, I, \mathcal {M}) = cert(q, I, \mathcal {M}^{\prime })$. In this case, we write $\mathcal {M} \equiv _{\mathsf {CQ}} \mathcal {M}^{\prime }$ (resp., $\mathcal {M} \equiv _{\mathsf {CQ}_{n}} \mathcal {M}^{\prime }$). The notion of *C* *Q*_*n*_-equivalence has been studied in the context of schema mapping optimization [5, 7]. Below we discuss its relationship to the convergence of schema mappings.

### **Proposition 3**

*Consider a sequence* $(\mathcal {M}_{n})_{n\geq 1}$ *of schema mappings and a schema mapping* $\mathcal {M}$.*Then* $\lim \limits _{n\to \infty }^{u} \mathcal {M}_{n} = \mathcal {M}$ *if and only if for every integer* *k* ≥ 1, *there is an integer* *n*_0_ ≥ 1*such that for all integers* *n* ≥ *n*_0_ *,* *we have that* $\mathcal {M}_{n} \equiv _{\mathsf {CQ}_{k}} \mathcal {M}$.

### *Proof 4*

The result follows by unfolding and comparing the definitions. Specifically, $\lim \limits _{n\to \infty }^{u} \mathcal {M}_{n} = \mathcal {M}$ means that for every *𝜖* > 0, there is an integer *n*_0_ ≥ 1 such that for every integer *n* ≥ *n*_0_ and for every source instance I we have that $dist(\text {Sol}(I,\mathcal {M}_{n}), \text {Sol}(I,\mathcal {M}))< \epsilon $. In turn, this means that for every integer *k* ≥ 1, there is an integer *n*_0_ ≥ 1 such that for every integer *n* ≥ *n*_0_ and for every source instance I we have that $\text {Sol}(I,M_{n}) \equiv _{\mathsf {CQ}_{k}} \text {Sol}(I,\mathcal {M})$. Thus, for every integer *k* ≥ 1, there is an integer *n*_0_ ≥ 1 such that for every integer *n* ≥ *n*_0_, we have that $\mathcal {M}_{n}\equiv _{\mathsf {CQ}_{k}}\mathcal {M}$. □

Intuitively, the preceding proposition states that it takes bigger and bigger conjunctive queries to distinguish the members of a sequence $(\mathcal {M}_{n})_{n\geq 1}$ from its uniform limit.

Although never explicitly introduced, the notion of uniform convergence was implicit in [5], where it was shown that for every SO tgd *σ* and for every *n* ≥ 1, there is a GLAV mapping $\mathcal {M}_{n}$ such that $\sigma \equiv _{\mathsf {CQ}_{n}} \mathcal {M}_{n}$. From this, it is easy to see that $\lim \limits _{n\to \infty }^{u} \mathcal {M}_{n} = \sigma $. Thus, we have the following result.

### **Theorem 1**

*(implicit in*[5]*)* *Every SO tgd is a uniform limit of a sequence of GLAV* *mappings.*

There are SO tgds that are not *C* *Q*-equivalent to any GLAV mapping. Indeed, from Example 4.6 and Theorem 4.10 in [7], it follows that the SO-tgd

$$\exists f\forall x \forall y(S(x,y) \rightarrow T(x,f(y))$$

is not *C* *Q*-equivalent to any GLAV mapping. Thus, the point of Theorem 1 is that SO tgds can be “approximated” up to any level of *C* *Q*_*k*_-equivalence by GLAV mappings, which are both syntactically simpler and generally more well-behaved.

As stated earlier, $(\mathcal {P}(\text {Inst}(\mathbf {T})), dist)$ is a pseudometric space since it cannot distinguish *C* *Q*-equivalent sets of instances. Consequently, the limit of a sequence of sets of instances and the (uniform or pointwise) limit of a sequence of mappings need not be unique. However, the limit is unique up to *C* *Q*-equivalence and, as described in Section 2, there is an associated metric space $(\widehat {\mathcal {P}(\text {Inst}(\mathbf {T}))}, \widehat {dist})$ obtained by considering the equivalence classes of $\mathcal {P}(\text {Inst}(\mathbf {T}))$ modulo the equivalence relation *R*_*dist*_, where $(\mathcal {J},\mathcal {J}^{\prime }) \in R_{dist}$ if and only if $dist(\mathcal {J},\mathcal {J}^{\prime }) = 0$ (i.e., if and only if $\mathcal {J} \equiv _{\mathsf {CQ}} \mathcal {J}^{\prime }$).

In subsequent sections, we will work with the metric space $(\widehat {\mathcal {P}(\text {Inst}(\mathbf {T}))}, \widehat {dist})$. Moreover, we will be interested in schema mappings modulo *C* *Q*-equivalence, which means that from now on we will view schema mappings as functions from source instances to equivalence classes of sets of target instances modulo *C* *Q*-equivalence. However, for notational simplicity, we will work each time with representatives of the equivalence classes. By a slight abuse of notation, we will write $(\mathcal {P}(\text {Inst}(\mathbf {T})),dist)$, instead of $(\widehat {\mathcal {P}(\text {Inst}(\mathbf {T}))}, \widehat {dist})$. Likewise, we will not explicitly distinguish between a schema mapping $\mathcal {M}$ and the equivalence class of the schema mappings that are *C* *Q*-equivalent to $\mathcal {M}$.

## Limits of Sequences of GAV Mappings

Our goal in this section is to analyze sequences of GAV mappings. To this effect, we first investigate the existence of limits of such sequences and then examine the definability of limits. As discussed in Section 3, if a sequence $(\mathcal {M}_{n})_{n\geq 1}$ of schema mappings has a pointwise (resp., uniform) limit, then the sequence is pointwise (resp., uniformly) Cauchy. The next result asserts that the converse holds for sequences of GAV mappings.

### **Theorem 2**

*Let* $(\mathcal {M}_{n})_{n\geq 1}$ *be a sequence of GAV mappings.*

- *If* $(\mathcal {M}_{n})_{n\geq 1}$ *is pointwise Cauchy, then it has a pointwise limit.*
- *If* $(\mathcal {M}_{n})_{n\geq 1}$ *is uniformly Cauchy, then it is eventually constant and thus has a GAV* *schema mapping as a uniform limit.*

### *Proof 5*

We consider GAV mappings over a source schema **S** and a target schema **T**. Let r denote the maximum arity of the relation symbols in **T**. For showing the first claim, assume that $(\mathcal {M}_{n})_{n\geq 1}$ is a pointwise Cauchy sequence of schema mappings and let I be a source instance. For each *n* ≥ 1, consider the universal solution $chase(I,\mathcal {M}_{n})$ for I w.r.t. $\mathcal {M}_{n}$ obtained by using the oblivious chase procedure. Since each $\mathcal {M}_{n}$ is a GAV schema mapping, we have that $chase(I,\mathcal {M}_{n})$ contains constants from the active domain of I and no nulls. We claim that there exists some *n*_0_ such that for all *n* ≥ *n*_0_, we have that $chase(I,\mathcal {M}_{n}) = chase(I, \mathcal {M}_{n_{0}})$. In other words, we claim that the sequence $(chase(I,\mathcal {M}_{n}))_{n\geq 1}$ is eventually constant (does not oscillate). Since every instance in the sequence $(chase(I,\mathcal {M}_{n}))_{n\geq 1}$ has no nulls, it can be identified by evaluating on that instance the atomic queries *R*(*x*_1_,…, *x*_*k*_), where R ranges over the relation symbols of **T** and k (with *k* ≤ *r*) denotes the arity of R. The assumption that the sequence $(chase(I,\mathcal {M}_{n}))_{n \geq 1}$ is pointwise Cauchy implies that there exists a positive integer *n*_0_ (that depends on I and r) such that for every integer *n* ≥ *n*_0_ and every conjunctive query *q* ∈*C* *Q*_*r*_, we have that $cert(q,I,\mathcal {M}_{n}) = cert(q,I,\mathcal {M})$. This implies that $q(chase(I,\mathcal {M}_{n})) = q(chase(I,\mathcal {M}_{n_{0}}))$ and, consequently, for every *n* ≥ *n*_0_, we have that $chase(I,\mathcal {M}_{n})) = chase(I,\mathcal {M}_{n_{0}})$.

We have *J*ust shown that if $(\mathcal {M}_{n})_{n\geq 1}$ is a pointwise Cauchy sequence of GAV mappings, then for every I, there exists a positive integer *m*_*I*_ such that $chase(I,\mathcal {M}_{m_{I}}) = chase(I,\mathcal {M}_{n})$, for all *n* ≥ *m*_*I*_. It follows that the schema mapping $\mathcal {M} = \{(I, chase(I,\mathcal {M}_{m_{I}}))\mid I \text { is a source instance}\}$ is a pointwise limit of the sequence $(\mathcal {M}_{n})_{n\geq 1}$. Note that $\mathcal {M}$ is indeed a schema mapping because $chase(I,\mathcal {M}_{m_{I}})$ contains no nulls.

For showing the second claim, assume that $(\mathcal {M}_{n})_{n\geq 1}$ is a uniformly Cauchy sequence of GAV mappings. We claim that $(\mathcal {M}_{n})_{n\geq 1}$ is eventually constant, i.e., there is some *n*_0_ such that for all *n* ≥ *n*_0_, $\mathcal {M}_{n}\equiv _{\mathsf {CQ}} \mathcal {M}_{n_{0}}$ holds. For this, we repeat the previous argument, but also note that, since the sequence $(\mathcal {M}_{n})_{n\geq 1}$ is uniformly Cauchy, there exists a positive integer *n*_0_ that depends only on r such that for every source instance I, for every integer *n* ≥ *n*_0_ and every conjunctive query *q* ∈*C* *Q*_*r*_, we have that $cert(q,I,\mathcal {M}_{n}) = cert(q,I,\mathcal {M})$. This implies that for every source instance I and every *n* ≥ *n*_0_, we have that $q(chase(I,\mathcal {M}_{n})) = q(chase(I,\mathcal {M}_{n_{0}}))$; consequently, for every source instance I and every *n* ≥ *n*_0_, we have that $chase(I,\mathcal {M}_{n})) = chase(I,\mathcal {M}_{n_{0}})$. □

Next, we point out that, for sequences of GAV mappings, the notions of pointwise convergence and uniform convergence are genuinely different.

### **Proposition 4**

*There exists a sequence of GAV mappings that has a GAV mapping as a* *pointwise limit, but has no uniform limit.*

### *Proof 6*

For every *n* ≥ 2, let $q_{n} = \bigwedge _{1 \leq i < j \leq n}(E(x_{i},x_{j}) \wedge E(x_{j},x_{i})).$ Intuitively, if E is interpreted as edge relation, then *q*_*n*_ yields a non-empty answer over any graph that contains a self-loop or a clique of size n. Let **S** be a source schema consisting of a binary relation symbol E and a unary relation symbol P, let **T** be a target schema consisting of a unary relation symbol *P* ^′^. Let $(\mathcal {M}_{n})_{n\geq 1}$ be the sequence of GAV mappings, where $\mathcal {M}_{n}$ is specified by the constraint ∀*x*∀*x*_1_,…, *x*_*n*+1_(*P*(*x*) ∧ *q*_*n*+1_ → *P* ^′^(*x*)). Intuitively, $\mathcal {M}_{n}$ is a “copy” schema mapping, but the copying action is triggered only if the source instance contains a self-loop or a clique of size *n* + 1. We will show that the GAV schema mapping $\mathcal {M} = \{\forall x\forall y(P(x) \wedge E(y,y) \rightarrow P^{\prime }(x))\}$ is a pointwise limit of $(\mathcal {M}_{n})_{n\geq 1}$, but that this pointwise limit is not a uniform limit of $(\mathcal {M}_{n})_{n\geq 1}$ and thus no uniform limit of $(\mathcal {M}_{n})_{n\geq 1}$ exists.

We first show that the GAV mapping $\mathcal {M}$ is a pointwise limit of $(\mathcal {M}_{n})_{n\geq 1}$. Given a source instance I, we consider two cases.

- If I contains a self-loop, then *J* = {*P* ^′^(*x*)∣*P*(*x*) ∈ *I*} is a universal solution for I w.r.t. $\mathcal {M}$ and w.r.t. $\mathcal {M}_{n}$, for all n. Thus, $cert(q,I,\mathcal {M}) = cert(q,I,\mathcal {M}_{n})$, for all n.
- If I is self-loop free, let *n*_0_ be such that no clique larger than *n*_0_ exists in I. Then, *J* = *∅* is a universal solution for I w.r.t. $\mathcal {M}$ and w.r.t. $\mathcal {M}_{n}$, for all *n* ≥ *n*_0_. Thus, $cert(q,I,\mathcal {M}) = cert(q,I,\mathcal {M}_{n})$, for all *n* ≥ *n*_0_.

Next, we show that $(\mathcal {M}_{n})_{n\geq 1}$ has no uniform limit. Towards a contradiction, suppose that such a uniform limit exists. Every uniform limit is also a pointwise limit; moreover, pointwise and uniform limits are unique up to *C* *Q*-equivalence. Hence, since the schema mapping $\mathcal {M}$ defined above is a pointwise limit of $(\mathcal {M}_{n})_{n\geq 1}$, it follows that $\mathcal {M}$ is also a uniform limit of $(\mathcal {M}_{n})_{n\geq 1}$. Let *m* = 1. Then there exists an *n*_0_ such that for all *n* ≥ *n*_0_ we have that $\mathcal {M}_{n} \equiv _{\mathsf {CQ}_{1}} \mathcal {M}$. Take *n* = *n*_0_. Let I be the source instance *K*_*n*+1_ ∪{*P*(*c*)} and let q be the target conjunctive query ∃*x* *P* ^′^(*x*). We now claim that $cert(q,I,\mathcal {M}_{n}) \neq cert(q,I,\mathcal {M})$, which contradicts the previously derived fact that $\mathcal {M}_{n} \equiv _{\mathsf {CQ}_{1}} \mathcal {M}$. Indeed, since I contains a clique of size *n* + 1, we have *P*(*c*) is a universal solution for I w.r.t. $\mathcal {M}_{n}$, hence $cert(q,I,\mathcal {M}_{n}) = \mathit {true}$. However, since I contains no self-loop, we have that *∅* is a universal solution for I w.r.t. $\mathcal {M}$, hence $cert(q,I,\mathcal {M}) = \mathit {false}$. □

Proposition 4 and Theorem 2 imply that the sequence of GAV mappings in the proof of Proposition 4 is an example of a pointwise Cauchy sequence that is not uniformly Cauchy. Theorem 2 also implies that if a sequence of GAV mappings has a uniform limit, then it must have a GAV mapping as such a limit. In turn, this gives rise to the following natural question concerning the definability of pointwise limits: if a sequence of GAV mappings has a pointwise limit, does it have a GAV mapping as such a limit? We answer this question in the negative by showing that even the much richer language of SO tgds cannot express pointwise limits of sequences of GAV mappings.

### **Proposition 5**

*There is a pointwise Cauchy sequence of GAV schema mappings such that* *no SO tgd is a pointwise limit of that sequence.*

### *Proof 7*

Consider a source schema **S** consisting of a binary relation symbol E, and a target schema **T** consisting of a binary relation F. For every *n* ≥ 1, let *P*_*n*_(*x*, *y*) be the conjunctive query expressing the property “there is an E-path of length n from x to y”, and let $\mathcal {M}_{n}$ be the GAV mapping specified by the set {∀*x*, *y*(*P*_*i*_(*x*, *y*) → *F*(*x*, *y*))∣1 ≤ *i* ≤ *n*}. Consider the schema mapping

$$\mathcal{M}^{\star} =\{(I, J) \mid F^{J} \text{ contains the transitive closure } TC(I) \text{ of } E^{I} \}.$$

It is easy to see that $\mathcal {M}^{\star }$ is a pointwise limit of the sequence $(\mathcal {M}_{n})_{n\geq 1}$; the reason for this is that, for every source instance *I* and for every *n* ≥|*a* *d* *o* *m*(*I*)|^2^, we have that $chase(I,\mathcal {M}_{n})= TC(I)$. However, $\mathcal {M}^{\star }$ is not *C* *Q*-equivalent to any schema mapping $\mathcal {M}^{\prime }$ that allows for *C* *Q*-rewriting: if it were, then there would exist a union *q* of conjunctive queries over the source such that, for every source instance *I*,

$$cert(F(x,y), I, \mathcal{M}^{*}) = TC(I) = cert(F(x,y), I, \mathcal{M}^{\prime}) = q(I).$$

Consequently, the transitive closure of *I* would be first-order definable over the source, which is not the case. Since every SO tgd allows for *C* *Q*-rewriting, no SO tgd is a pointwise limit of the sequence $(\mathcal {M}_{n})_{n\geq 1}$.□

We have just seen that there are sequences of GAV mappings that have a pointwise limit, but no such limit is definable by a GAV mapping. This raises the question of finding necessary and sufficient conditions guaranteeing that a sequence of GAV mappings has a GAV mapping as a pointwise limit. The next result provides an answer to this question.

### **Theorem 3**

*Let* $(\mathcal {M}_{n})_{n\geq 1}$ *be a pointwise Cauchy sequence of GAV mappings. The following statements are* *equivalent:*

1. $(\mathcal {M}_{n})_{n\geq 1}$ *has a GAV mapping as a pointwise limit.*
2. $(\mathcal {M}_{n})_{n\geq 1}$ *has a pointwise limit that allows for* *C* *Q* *-rewriting.*

### *Proof 8*

Let $(\mathcal {M}_{n})_{n\geq 1}$ be a pointwise Cauchy sequence of schema mappings. As shown in the proof of Theorem 2, for every source instance I, there is a positive integer *m*_*I*_, such that for all *n* ≥ *m*_*I*_ the equality $chase(I, \mathcal {M}_{m_{I}}) = chase(I, \mathcal {M}_{n})$ holds for the respective elements $\mathcal {M}_{m_{I}}$ and $\mathcal {M}_{n}$ of $(\mathcal {M}_{n})_{n\geq 1}$. Moreover, the schema mapping

$$\mathcal{M} = \{(I,\text{ chase}(I,\mathcal{M}_{m_{I}})) \mid\, I\text{ is a source instance} \} $$

is a pointwise limit of $(\mathcal {M}_{n})_{n\geq 1}$. Consider the following schema mapping $\mathcal {M}^{\star }$:

$$\mathcal{M}^{\star}= \{(I,J) \mid\text{ chase}(I,\mathcal{M}_{m_{I}}) \subseteq J\} $$

It is clear that $\mathcal {M}^{\star }$ is also a pointwise limit of $(\mathcal {M}_{n})_{n\geq 1}$. The result we seek is an immediate consequence of the fact that the following four statements are equivalent:

- $(\mathcal {M}_{n})_{n\geq 1}$ has a GAV mapping as a pointwise limit.
- $(\mathcal {M}_n)_{n\geq 1}$ has a pointwise limit that allows for *C* *Q*-rewriting.
- $\mathcal {M}^{\star }$ allows for *C* *Q*-rewriting.
- $\mathcal {M}^{\star }$ is logically equivalent to a GAV mapping.

We now show that these four conditions are equivalent.

- (a) ⇒ (b) This is true because every GAV mapping allows for *C* *Q*-rewriting.
- (b) ⇒ (c) This is true because if $\mathcal {M}^{\prime }$ is a pointwise limit of $(\mathcal {M}_{n})_{n\geq 1}$ that allows for *C* *Q*-rewriting, then so does $\mathcal {M}^{\star }$ since ${\mathcal {M}^{\prime }} \equiv _{\mathsf {CQ}} \mathcal {M}^{\star }$.
- (c) ⇒ (d) This is the most involved part of the proof. Let us examine the structural properties that the schema mapping $\mathcal {M}^{\star }$ possesses. By hypothesis, $\mathcal {M}^{\star }$ allows for *C* *Q*-rewriting. By construction, $\mathcal {M}^{\star }$ admits universal solutions, since $chase(I,\mathcal {M}_{m_{I}})$ is a universal solution for *I* w.r.t. $\mathcal {M}^{\star }$, for every source instance *I*. Moreover, it is clear from its definition that $\mathcal {M}^{\star }$ is closed under target homomorphisms. Finally, we claim that $\mathcal {M}^{\star }$ is closed under target intersections. Indeed, assume that both (*I*, *J*_1_) and (*I*, *J*_2_) are in $\mathcal {M}^{\star }$. Then $chase(I,\mathcal {M}_{m_{I}})$ is contained in both *J*_1_ and *J*_2_, hence $chase(I,\mathcal {M}_{m_{I}})$ is contained in *J*_1_ ∩ *J*_2_, hence *J*_1_ ∩ *J*_2_ is a solution for *I* w.r.t. $\mathcal {M}^{\star }$.

Thus, $\mathcal {M}^{\star }$ allows for *C* *Q*-rewriting, admits universal solutions, and is closed under both target homomorphisms and target intersections. Theorem 3.2 in [19] asserts that a schema mapping is logically equivalent to a GAV schema mapping if and only if it allows for *C* *Q*-rewriting, admits universal solutions, and is closed under both target homomorphisms and target intersections. It follows that $\mathcal {M}^{\star }$ is logically equivalent to a GAV mapping.

- (d) ⇒ (a) This is obvious since $\mathcal {M}^{\star }$ is a pointwise limit of $(\mathcal {M}_n)_{n\geq 1}$.

□

Observe that Theorem 3 (and its proof) provide necessary and sufficient conditions for a pointwise Cauchy sequence of GAV mappings to have a GAV mapping as a pointwise limit, but these conditions are on the pointwise limit and not on the sequence itself. By analyzing the proof of Theorem 3, however, it is possible to extract a necessary and sufficient condition on the sequence itself. For this, we need to introduce the following concept.

### **Definition 6**

Let $(\mathcal {M}_{n})_{n\geq 1}$ be a sequence of schema mappings. We say that $(\mathcal {M}_{n})_{n\geq 1}$ *allows for* *C* *Q* *-rewriting* if for every target conjunctive query q, there is a union *q* ^′^ of source conjunctive queries having the following property: for every source instance I, there is a positive integer *n*_*I*_ such that $cert(q,I,\mathcal {M}_{n}) = q^{\prime }(I)$, for every *n* ≥ *n*_*I*_.

Let $\mathcal {M}$ be a pointwise limit of a sequence $(\mathcal {M}_{n})_{n\geq 1}$ of schema mappings. It is easy to show that $\mathcal {M}$ allows for *C* *Q*-rewriting if and only if $(\mathcal {M}_{n})_{n\geq 1}$ allows for *C* *Q*-rewriting. Indeed, assume first that $\mathcal {M}$ allows for *C* *Q*-rewriting. To show that $(\mathcal {M}_{n})_{n\geq 1}$ allows for *C* *Q*-rewriting, let *q* be a conjunctive query and let *q* ^′^ be a union of conjunctive queries such that $cert(q,I,\mathcal {M}) = q^{\prime }(I)$, for every source instance *I*. Since $\mathcal {M}$ is a pointwise limit of $(\mathcal {M}_{n})_{n\geq 1}$, for every instance *I*, there is a positive integer $n^{\prime }_{I}$ such that $cert(q,I,\mathcal {M}) = cert(q,I,\mathcal {M}_{n})$, for every $n\geq n^{\prime }_{I}$. It follows that $cert(q,I,\mathcal {M}_{n}) = q^{\prime }(I)$, for every $n\geq n^{\prime }_{I}$, which shows that $(\mathcal {M}_{n})_{n\geq 1}$ allows for *C* *Q*-rewriting. In the other direction, assume that $(\mathcal {M}_{n})_{n\geq 1}$ allows for *C* *Q*-rewriting. To show that $\mathcal {M}$ allows for *C* *Q*-rewriting, let *q* be a conjunctive query and let *q* ^′^ be a union of conjunctive queries such that for every source instance *I*, there is a positive integer *n*_*I*_ such that $cert(q,I,\mathcal {M}_{n}) = q^{\prime }(I)$, for every *n* ≥ *n*_*I*_. By the pointwise convergence of $(\mathcal {M}_{n})_{n\geq 1}$ to $\mathcal {M}$, for every source instance *I*, there is a positive integer $n^{\prime }_{I}$ such that $cert(q,I,\mathcal {M}) = cert(q,I,\mathcal {M}_{n})$, for every $n\geq n^{\prime }_{I}$. Let *I* be a source instance. By taking any $n\geq \max \{n_{I},n^{\prime }_{I}\}$, we have that $cert(q,I,\mathcal {M}) = cert(q,I,M_{n})$ and *c* *e* *r* *t*(*q*, *I*, *M*_*n*_) = *q* ^′^(*I*), hence $cert(q,I,\mathcal {M}) = q^{\prime }(I)$, which shows that $\mathcal {M}$ allows for *C* *Q*-rewriting.

By combining the preceding remarks with Theorems 2 and 3, we obtain the following result.

### **Corollary 1**

*Let* $(\mathcal {M}_{n})_{n\geq 1}$ *be a pointwise Cauchy sequence of GAV mappings. The following statements are* *equivalent:*

1. $(\mathcal {M}_{n})_{n\geq 1}$ *has a GAV mapping as a pointwise limit.*
2. $(\mathcal {M}_{n})_{n\geq 1}$ *allows for* *C* *Q* *-rewriting.*

Since every schema mapping specified by an SO tgd allows for *C* *Q*-rewriting, Theorem 3 also implies the following result.

### **Corollary 2**

*Let* $(\mathcal {M}_{n})_{n\geq 1} $ *be a pointwise Cauchy sequence of GAV mappings. The following statements are* *equivalent:*

1. $(\mathcal {M}_{n})_{n\geq 1}$ *has a GAV mapping as a pointwise limit.*
2. $(\mathcal {M}_{n})_{n\geq 1}$ *has an SO tgd as a pointwise limit.*

Finally, we note that Proposition and Theorem 3 yield a fairly complete picture of the definability of pointwise limits of GAV mappings. Specifically, there are two mutually exclusive possibilities:

1. No pointwise limit allows for *C* *Q*-rewriting and no GAV mapping is a pointwise limit.
2. Every pointwise limit admits *C* *Q*-rewriting and there is a GAV mapping that is a pointwise limit. Moreover, this happens precisely when the schema mapping $\mathcal {M}^{\star }$ in the proof of Theorem 3 allows for *C* *Q*-rewriting or, equivalently, when $\mathcal {M}^{\star }$ is logically equivalent to a GAV mapping.

## Limits of Sequences of LAV Mappings

In this section, we investigate the existence and definability of limits of sequences of LAV mappings. In fact, we will consider a much broader class of GLAV mappings, namely *k-premise-bounded* GLAV mappings for arbitrary *k* ≥ 1. LAV mappings correspond to the special case of *k* = 1.

### **Definition 7**

Let $\mathcal {M}$ be a GLAV mapping and k a positive integer. We call $\mathcal {M}$ a k-premise-bounded GLAV mapping if the premise of every constraint in $\mathcal {M}$ has at most k atoms.

Let $(\mathcal {M}_{n})_{n\geq 1}$ be a sequence of GLAV mappings. We say that $(\mathcal {M}_{n})_{n\geq 1}$ is premise-bounded if there exists an integer k such that every element $\mathcal {M}_{n}$ of $(\mathcal {M}_{n})_{n\geq 1}$ is k-premise bounded.

Unlike the case of GAV mappings, the notions of pointwise Cauchy and uniformly Cauchy sequences of premise-bounded GLAV mappings coincide. Moreover, the same holds true for the notions of pointwise limit and uniform limit of sequences of such schema mappings.

### **Theorem 4**

*Let* $(\mathcal {M}_{n})_{n\geq 1}$ *be a sequence of premise-bounded GLAV mappings.*

1. *The sequence* $(\mathcal {M}_{n})_{n\geq 1}$ *is pointwise Cauchy if and only if it is uniformly Cauchy.*
2. *The sequence* $(\mathcal {M}_{n})_{n\geq 1}$ *has a pointwise limit if and only if it has a uniform limit.*

### *Proof 9*

We prove the first part and then use it to prove the second part.

**Part 1**. It is obvious that every uniformly Cauchy sequence of mappings is also pointwise Cauchy. We focus on the reverse direction. Let $(\mathcal {M}_{n})_{n\geq 1}$ be a pointwise Cauchy sequence of premise bounded GLAV mappings. We have to show that for every m, there is an *N*_0_ such that for all *n*, *n* ^′^≥ *N*_0_, we have that $\mathcal {M}_{n} \equiv _{\mathsf {CQ}_{m}} \mathcal {M}_{n^{\prime }}$.

Fix an integer m. Since $(\mathcal {M}_{n})_{n\geq 1}$ is pointwise Cauchy, for every source instance I, there is an integer *n*_0_(*I*) such that for all *n*, *n* ^′^≥ *n*_0_(*I*) and for every conjunctive query q in *C* *Q*_*m*_, we have that $cert(q,I,\mathcal {M}_{n}) = cert(q,I,\mathcal {M}_{n^{\prime }})$. Let p be the number of relation symbols in the target schema, let r be their maximum arity, and let k be the bound on the number of atoms in the premises of the members of the sequence $(\mathcal {M}_{n})_{n\geq 1}$. We write $\mathcal {I}$ to denote the class of all source instances with at most *k* ⋅ *p* ⋅ *m* ^*r*^ atoms. Clearly, up to isomorphism, there are only finitely many instances $I \in \mathcal {I}$. Moreover, if *I* ^′^≅*I* ^″^, then *n*_0_(*I* ^′^) = *n*_0_(*I* ^″^). Consequently, the quantity $N_{0} = \mathop {max}\{n_{0}(I) \mid I \in \mathcal {I}\}$ is a positive integer. We claim that for all *n*, *n* ^′^≥ *N*_0_, we have that $\mathcal {M}_{n} \equiv _{\mathsf {CQ}_{m}} \mathcal {M}_{n^{\prime }}$.

Let I be an arbitrary source instance and let q be an arbitrary conjunctive query in *C* *Q*_*m*_. We have to show that $cert(q,I, \mathcal {M}_{n}) = cert(q, I, \mathcal {M}_{n^{\prime }})$, for all *n*, *n* ^′^≥ *N*_0_. Let **a** be a tuple of constants such that $\mathbf {a} \in cert(q, I, \mathcal {M}_{n})$, hence $\mathbf {a} \in q(chase(I, \mathcal {M}_{n}))_{\downarrow }$. Since the query q has at most m variables, it must consist of at most *p* ⋅ *m* ^*r*^ atoms. Let $h: \mathit {atoms}(q) \to chase(I, \mathcal {M}_{n})$ be a homomorphism establishing that $\mathbf {a} \in q(chase(I,\mathcal {M}_{n}))$. It follows that there are at most *p* ⋅ *m* ^*r*^ facts in $chase(I,\mathcal {M}_{n})$ witnessing that $\mathbf {a} \in q(chase(I, \mathcal {M}_{n}))_{\downarrow }$. Each of these facts must be produced in a single step while chasing the source instance I with $\mathcal {M}_{n}$, which implies that each of these facts is produced using at most k facts from I. Let *I* ^∗^ be the subinstance of I consisting of all the aforementioned facts of I used to produce the facts in $chase(I,\mathcal {M}_{n})$ witnessing that $\mathbf {a} \in q(chase(I, \mathcal {M}_{n}))_{\downarrow }$. We then have that |*I* ^∗^|≤ *k* ⋅ *p* ⋅ *m* ^*r*^ and $\mathbf {a} \in q(chase(I^{*}, \mathcal {M}_{n}))_{\downarrow }$. Since *n*, *n* ^′^≥ *N*_0_, we have that $q(chase(I^{*}, \mathcal {M}_{n}))_{\downarrow } = q(chase(I^{*}, \mathcal {M}^{\prime }_{n}))_{\downarrow }$, hence $\mathbf {a} \in q(chase(I^{*}, \mathcal {M}^{\prime }_{n}))_{\downarrow }$. By the monotonicity of the chase procedure, we have that $\mathbf {a} \in q(chase(I, \mathcal {M}^{\prime }_{n}))_{\downarrow }$. It follows that $q(chase(I, \mathcal {M}_{n}))_{\downarrow } \subseteq q(chase(I, \mathcal {M}^{\prime }_{n}))_{\downarrow }$. A symmetric argument establishes the containment $q(chase(I, \mathcal {M}^{\prime }_{n}))_{\downarrow } \subseteq q(chase(I, \mathcal {M}_{n}))_{\downarrow }$, hence $q(chase(I, \mathcal {M}_{n}))_{\downarrow } = q(chase(I, \mathcal {M}^{\prime }_{n}))_{\downarrow }$, which, in turn, implies that $cert(q,I, \mathcal {M}_{n}) = cert(q, I, \mathcal {M}_{n^{\prime }})$.

**Part 2**. It is obvious that if a sequence of schema mappings has a uniform limit, then it has a pointwise limit. We focus on the reverse direction. Let $(\mathcal {M}_{n})_{n\geq 1}$ be a sequence of premise bounded GLAV mappings that has a pointwise limit $\mathcal {M}$. We claim that $\mathcal {M}$ is also a uniform limit of $(\mathcal {M}_{n})_{n\geq 1}$.

Since $(\mathcal {M}_{n})_{n\geq 1}$ has a pointwise limit, we have that $(\mathcal {M}_{n})_{n\geq 1}$ is pointwise Cauchy. The previous part implies that $(\mathcal {M}_{n})_{n\geq 1}$ is uniformly Cauchy as well. Fix an integer m. Since $(\mathcal {M}_{n})_{n\geq 1}$ is uniformly Cauchy, there exists an *n*_0_ such that for all *n*, *n* ^′^≥ *n*_0_, we have that $\mathcal {M}_{n} \equiv _{\mathsf {CQ}_{m}} \mathcal {M}_{n^{\prime }}$. We claim that also $\mathcal {M}_{n} \equiv _{\mathsf {CQ}_{m}} \mathcal {M}$ holds, for every *n* ≥ *n*_0_. To show this, fix some *n* ≥ *n*_0_ and let I be a source instance and q a conjunctive query in *C* *Q*_*m*_. We have to show that $cert(q,I,\mathcal {M}_{n}) = cert(q,I,\mathcal {M})$. Since $\mathcal {M}$ is a pointwise limit of $(\mathcal {M}_{n})_{n\geq 1}$, there is an $n^{\prime }_{0}(I)$ such that for all $n^{\prime } \geq n^{\prime }_{0}(I)$, we have that $cert(q,I,\mathcal {M}_{n^{\prime }})=cert(q,I,\mathcal {M})$. Take an integer *n* ^′^ such that $n^{\prime } \geq \max \{n_{0}, n^{\prime }_{0}(I)\}$. Since *n* ^′^≥ *n*_0_, we have that $cert(q,I,\mathcal {M}_{n}) = cert(q,I,\mathcal {M}_{n^{\prime }})$. Since $n^{\prime }\geq n^{\prime }_{0}(I)$, we have that $cert(q,I,\mathcal {M}_{n^{\prime }}) = cert(q,I,\mathcal {M})$. Thus, $cert(q,I,\mathcal {M}_{n}) = cert(q,I,\mathcal {M})$. □

Note that the preceding proof of Part 2 used only the hypothesis that the sequence $(\mathcal {M}_{n})_{n\geq 1}$ is uniformly Cauchy and the fact that the sequence $(\mathcal {M}_{n})_{n\geq 1}$ has a pointwise limit, as we have proved in Part 1. As a matter of fact, this is an instance of a general result about pseudometric spaces, namely, that if a uniformly Cauchy sequence of functions converges pointwise, then it also converges uniformly.

The following two propositions further demarcate the differences between GAV and premise-bounded GLAV mappings. In fact, these differences are already witnessed by sequences of LAV mappings. The first difference concerns the existence of limits of uniformly Cauchy sequences. In contrast to the GAV case, uniformly Cauchy sequences of LAV mappings may have no uniform limit; in fact, they may not even have a pointwise limit.

### **Proposition 6**

*There exists a uniformly Cauchy sequence of LAV mappings that has no* *pointwise limit; in particular, it has no uniform limit either.*

### *Proof 10*

Let **S** be a source schema consisting of a binary relation symbol E and let **T** be a target schema consisting of a binary relation F. For every *n* ≥ 1, let $\mathcal {M}_{n}$ be the LAV mapping specified by the constraint

$$\forall x,y (E(x,y) \to q_{n+1})$$

where $q_{n} = \exists z_{1},{\ldots } z_{n} \bigwedge _{1 \leq i < j \leq n}(F(z_{i},z_{j}) \wedge F(z_{j},z_{i}))$ is the boolean conjunctive query which is satisfied by the graphs containing a self-loop or a clique of size *n* (now considering *F* as the edge relation).

We first show that the sequence$(\mathcal {M}_{n})_{n\geq 1}$ is uniformly Cauchy. Let *k* ≥ 1. We claim that if we take *n*_0_ = *k*, then for every source instance *I*, for every *n*, *m* ≥ *n*_0_, and every *q* ∈*C* *Q*_*k*_, we have that $cert(q, I, \mathcal {M}_{n}) = cert(q, I, \mathcal {M}_{m})$. To see this, note that for every source instance *I* and for every *t* ≥ 1, the universal solutions of *I* w.r.t. $\mathcal {M}_{t}$ have active domains consisting entirely of labeled nulls. Hence, only boolean queries may return a non-empty result. Moreover, observe that these universal solutions have no self-loops, i.e., they contain no atoms of the form *F*(*v*, *v*) for some labeled null *v*.

We now distinguish two cases: First, suppose that *q* ∈*C* *Q*_*k*_ is a boolean conjunctive query which contains a “self-loop”, i.e., an atom of the form *F*(*z*, *z*) for some variable *z*. Then we clearly have $cert(q, I, \mathcal {M}_{n}) = \mathit {false} = cert(q, I, \mathcal {M}_{m})$. It remains to consider the case that *q* ∈ *C* *Q*_*k*_ is a boolean *C* *Q* containing no self-loop. Then we clearly have $cert(q, I, \mathcal {M}_{n}) = \mathit {true} = cert(q, I, \mathcal {M}_{m})$, since we are assuming that *m*, *n* ≥ *k* holds.

Using an argument similar to the one in the proof of Proposition 2, we now show that the sequence $(\mathcal {M}_{n})_{n\geq 1}$ has no pointwise limit. Towards a contradiction, assume that $(\mathcal {M}_{n})_{n\geq 1}$ does have a pointwise limit $\mathcal {M}$. Let *I* be a non-empty source instance. We consider three cases.

First, assume that $\text {Sol}(I,\mathcal {M})$ is empty. Then, for every boolean conjunctive query *q*, it holds trivially that $cert(q,I,\mathcal {M}) = \mathit {true}$. This is, in particular, the case for the query *q* = ∃*z* *F*(*z*, *z*), which asks for the existence of a self-loop. However, for this query *q*, we have that $cert(q, I, \mathcal {M}_{n}) = \mathit {false}$ for every *n* ≥ 1.

Second, assume that $\text {Sol}(I,\mathcal {M})$ is non-empty and that all solutions $J \in \text {Sol}(I,\mathcal {M})$ contain a self-loop. For the query *q* = ∃*z* *F*(*z*, *z*) as above, we again have $cert(q, I, \mathcal {M}) = {\mathit {true}}$, whereas $cert(q, I, \mathcal {M}_{n}) = \mathit {false}$, for every *n* ≥ 1.

Finally, assume that $\text {Sol}(I,\mathcal {M})$ is non-empty and that at least one solution $J \in \text {Sol}(I,\mathcal {M})$ does not contain a self-loop. Let *m* be the biggest integer such that *J* contains a clique of size *m*. Consider the conjunctive query

$$q = \exists z_{1}, {\dots} z_{m+1} \bigwedge\limits_{1 \leq i < j \leq m+1} (F(z_{i},z_{j}) \wedge F(z_{j},z_{i})).$$

Then *q* evaluates to false over *J* and we have $cert(q, I, \mathcal {M}) = \mathit {false}$. On the other hand, for all *n* ≥ *m* + 1 we have $cert(q, I, \mathcal {M}_{n}) = \mathit {true}$. Again, this contradicts our assumption that $\mathcal {M}$ is the pointwise limit of $(\mathcal {M}_{n})_{n\geq 1}$. □

The next difference is the definability of uniform limits. In Section 4, we saw that if a sequence of GAV mappings has a uniform limit, then it is eventually constant, hence it has a GAV mapping as a uniform limit. This property need not hold for sequences of LAV mappings (hence, it need not hold for sequences of premise-bounded schema mappings).

### **Proposition 7**

*There exists a sequence* $(\mathcal {M}_{n})_{n\geq 1}$ *of LAV mappings that has a uniform limit, but no uniform limit of* $(\mathcal {M}_{n})_{n\geq 1}$ *admits universal solutions. In particular, no* *SO tgd is a uniform limit of the sequence* $(\mathcal {M}_{n})_{n\geq 1}$.

### *Proof 11*

For every *n* ≥ 1, let $\mathcal {M}_{n}$ be the LAV mapping specified by the constraint

$$\forall x (V(x) \to \exists P_{n})$$

where ∃*P*_*n*_ is the conjunctive query ∃*z*_1_…∃*z*_*n*_(*F*(*z*_1_, *z*_2_) ∧… ∧ *F*(*z*_*n*−1_, *z*_*n*_)) asserting that there is a “path” (possibly with repeated vertices) of length *n* in the target instance. We now show that the sequence $(\mathcal {M}_{n})_{n\geq 1}$ has a uniform limit, but no uniform limit of this sequence admits universal solutions.

**Part 1**. For the first part of the claim, consider the schema mapping

$$\mathcal{M} = \{(I, J) \mid I\neq \emptyset\text{ and }J\in v(C_{k}), k > 1\},$$

where *C*_*k*_ is a target instance consisting of a simple cycle of nulls of size *k* and *v*(*C*_*k*_) is the set of all isomorphic copies of *C*_*k*_ via isomorphisms that rename nulls. We will show that $\mathcal {M}$ is a uniform limit of the sequence $(\mathcal {M}_{n})_{n\geq 1}$. Specifically, we will show for every *m*, there exists *n*_0_ such that for all *n* ≥ *n*_0_, we have that $\mathcal {M}_{n} \equiv _{\mathsf {CQ}_{m}} \mathcal {M}$.

Let *n*_0_ = *m*. Since each $\mathcal {M}_{n}$ has solutions consisting entirely of nulls, it suffices to consider boolean *C* *Q* s only. Let *q* be a boolean *C* *Q* with *m* variables and assume that $cert(q, I, \mathcal {M}_{n})= true$, where *n* ≥ *m*. This implies that there is a homomorphism from the body of *q* into *P*_*n*_, where *P*_*n*_ is the simple path with *n* nodes. In turn, this implies that *C*_*k*_⊧*q*, for every *k*. Thus, $cert(q, I, \mathcal {M}) = \mathit {true}$ as well. In the other direction, assume that $cert(q, I, \mathcal {M}) = \mathit {true}$. Note that *q* cannot contain a directed cycle, since no directed cycle can be mapped homomorphically in *every* cycle of length greater than one. Let *h* be a homomorphism from the body of *q* into *C*_*m*+1_. Since *q* ∈*C* *Q*_*m*_, the variables of *q* have at most *m* distinct images among the nodes of *C*_*m*+1_. This means that $\tilde C_{m+1} \models q$, where $\tilde C_{m+1}$ is obtained from *C*_*m*+1_ by removing the facts that contain at least one element that is not the image of one of the variables of *q* under *h*. Note that $\tilde C_{m+1}$ has at least one fact less than *C*_*m*+1_, and so it is a collection of simple paths of length at most *m*; therefore, there is a homomorphism from $\tilde C_{m+1}$ to *P*_*n*_, hence *P*_*n*_⊧*q*.

**Part 2**. For the second part of the claim and towards a contradiction, assume that $\mathcal {M}^{\prime }$ is a uniform limit of $(\mathcal {M}_{n})_{n\geq 1}$ such that there exists a non-empty source instance *I* and a finite universal solution *J* for *I* w.r.t. $\mathcal {M}^{\prime }$. Note that for every *i*, we have that $cert(\exists P_{i}, I, \mathcal {M}^{\prime }) = \mathit {true}$, because $\mathcal {M}^{\prime }$ is a (uniform and, hence also pointwise) limit of the sequence $(\mathcal {M}_{n})_{n\geq 1}$. Then we also have that *J*⊧∃*P*_*i*_, since *J* is universal. Since *J* is finite, this is possible only if *J* contains a directed cycle.

We can now derive a contradiction as follows. For each positive integer *l*, let ∃*C*_*l*_ be the boolean conjunctive query asserting the existence of a cycle of length *l*. Then there is no *n* such that $cert(\exists C_{l}, I, \mathcal {M}_{n}) = \mathit {true}$. Thus, $cert(\exists C_{l}, I, \mathcal {M}^{\prime }) = \mathit {false}$ must hold for every *l*, since $\mathcal {M}^{\prime }$ is a limit of $(\mathcal {M}_{n})_{n\geq 1}$. Hence, *J* cannot contain cycles.

Since every SO tgd admits universal solutions, it follows that no SO tgd is a (uniform or pointwise) limit of $(\mathcal {M}_{n})_{n\geq 1}$. □

By Theorem 1, every SO tgd is the uniform limit of a sequence of GLAV mappings. Proposition 7 implies that the converse is false, even for sequences of LAV mappings.

In the previous section, we showed that a sequence of GAV mappings has a GAV mapping as a pointwise limit if and only if it has a pointwise limit that allows for *C* *Q*-rewriting. Is there some structural property that characterizes when a sequence of premise-bounded GLAV mappings has a GLAV mapping as a pointwise limit (which, for premise-bounded mappings, is the same as a uniform limit)? We will show that the property of admitting universal solutions is the key to this question. Specifically, we have the following result.

### **Theorem 5**

*Let* $(\mathcal {M}_{n})_{n\geq 1}$ *be a premise-bounded sequence of GLAV mappings. The following statements are* *equivalent.*

1. $(\mathcal {M}_{n})_{n\geq 1}$ *has a GLAV mapping* $\mathcal {M}$ *as a uniform limit.*
2. $(\mathcal {M}_{n})_{n\geq 1}$ *has a uniform limit that admits universal solutions.*

*Moreover, if* $(\mathcal {M}_{n})_{n\geq 1}$ *is a sequence* *of LAV mappings, then* $(\mathcal {M}_{n})_{n\geq 1}$ *has a LAV mapping as a uniform limit if and only* $(\mathcal {M}_{n})_{n\geq 1}$ *has a* *uniform limit that admits universal solutions.*

We now give two lemmas which will be used in the proof of Theorem 5, but are also of interest in their own right.

### **Lemma 2**

*If* $\mathcal {M}$ *is the uniform limit of a sequence* $(\mathcal {M}_{n})_{n\geq 1}$ *of schema mappings each of which allows for* *C* *Q* *-rewriting,* *then also* $\mathcal {M}$ *allows for* *C* *Q* *-rewriting.*

### *Proof 12*

Let q be a target conjunctive query with m variables. Since $\mathcal {M}$ is a uniform limit of $(\mathcal {M}_{n})_{n\geq 1}$, there exists an integer *n*_0_ such that for every *n* ≥ *n*_0_ and every source instance I, we have that $cert(q,I,\mathcal {M}) = cert(q,I,\mathcal {M}_{n})$. In particular, $cert(q,I,\mathcal {M}) = cert(q,I,\mathcal {M}_{n_{0}})$. Since $\mathcal {M}_{n_{0}}$ allows for *C* *Q*-rewriting, there is a source conjunctive query *q* ^′^ such that $cert(q,I,\mathcal {M}_{n_{0}}) = q^{\prime }(I)$, for every source instance I. Hence, $cert(q,I,\mathcal {M}) = q^{\prime }(I)$ holds, for every source instance I. □

It should be noted that the conclusion of Lemma 2 does not hold, in general, if $\mathcal {M}$ is a pointwise limit of a sequence $(\mathcal {M}_{n})_{n\geq 1}$ of schema mappings each of which allows for *C* *Q*-rewriting. Indeed, if $(\mathcal {M}_{n})_{n\geq 1}$ is the sequence of GAV mappings in the proof of Proposition 5, then Theorem 3 and Proposition 5 imply that no pointwise limit of $(\mathcal {M}_{n})_{n\geq 1}$ allows for *C* *Q*-rewriting.

### **Lemma 3**

*Let* $\mathcal {M}$ *be a uniform limit of a sequence* $(\mathcal {M}_{n})_{n\geq 1}$ *of LAV mappings. If* $\mathcal {M}$ *admits universal solutions, then it is closed under unions.*

### *Proof 13*

The proof proceeds through several stages and involves four claims, each of which builds on preceding ones. We first state the claims without proof and then use the last claim to show the desired conclusion. After this, we complete the proof of the lemma by proving each claim.

We first modify the notion of *C* *Q*-equivalence by limiting the number of atoms of *C* *Q* s, rather than the number of variables. This yields an equivalent notion of uniform limit.

- For *ℓ* ≥ 1, we define *C* *Q* ^′^_*ℓ*_ = {*q* ∈*C* *Q* ∣ *l* *e* *n* *g* *t* *h*(*q*) ≤ *ℓ*}, where *l* *e* *n* *g* *t* *h*(*q*) denotes the number of atoms in q.
- We say that two schema mappings $\mathcal {M}_{1}$ and $\mathcal {M}_{2}$ are *C* *Q* ^′^_*ℓ*_-equivalent, denoted by $\mathcal {M}_{1} \equiv _{\mathsf {CQ}^{\prime }_{\ell }} \mathcal {M}_{2}$, if for every source instance *I* and for every $q \in \mathsf {CQ}^{\prime }_{\ell }$, we have that $cert(q,I,\mathcal {M}_{1}) = cert(q,I,\mathcal {M}_{2})$.
- We say that $\mathcal {M}$ is the *u* ^′^-limit of a sequence $(\mathcal {M}_{n})_{n\geq 1}$, denoted by $\mathcal {M}_{n} \stackrel {u^{\prime }}{\longrightarrow } \mathcal {M}$, if for every *ℓ*, there exists *n*_0_ such that for all *n* ≥ *n*_0_, it holds that $\mathcal {M}_{n} \equiv _{\mathsf {CQ}^{\prime }_{\ell }} \mathcal {M}$.

### **Claim A.**

The notions of *u* ^′^-limit and uniform limit coincide. Formally, for every sequence $(\mathcal {M}_{n})_{n\geq 1}$ of schema mappings and every schema mapping $\mathcal {M}$, we have that $\mathcal {M}_{n} \stackrel {u}{\longrightarrow } \mathcal {M}$ if and only if $\mathcal {M}_{n} \stackrel {u^{\prime }}{\longrightarrow } \mathcal {M}$.

Next, we use the given sequence $(\mathcal {M}_{n})_{n\geq 1}$ to construct another sequence $(\mathcal {M}^{\prime }_{n})_{n\geq 1}$ of LAV mappings that possesses some desirable properties. To define the sequence $(\mathcal {M}^{\prime }_{n})_{n\geq 1}$, we need another claim.

### **Claim B.**

Assume that $\mathcal {M}_{n} \stackrel {u}{\longrightarrow } \mathcal {M}$. Then, there exists a strictly increasing sequence (*n*_*i*_)_*i* ≥ 1_ of positive integers, such that for every *ℓ* ≥ 1 and for every *n* ≥ *n*_*ℓ*_, we have that $\mathcal {M}_{n} \equiv _{\mathsf {CQ}^{\prime }_{\ell }} \mathcal {M}$.

Let (*n*_*i*_)_*i* ≥ 1_ be the strictly increasing sequence of positive integers according to Claim B. We define the sequence $(\mathcal {M}^{\prime }_{n})_{n\geq 1}$ of LAV mappings as follows:

$$\mathcal{M}^{\prime}_{n} = \left\{\begin{array}{ll} \mathcal{M}_{n} & \text{if } n < n_{1} \\ \displaystyle\bigcup\limits_{\tau \in \mathcal{M}_{n}} T(\tau,\ell) & \text{otherwise, if } n_{\ell} \leq n < n_{\ell +1} \end{array}\right. $$

Here, *T*(*τ*, *ℓ*) contains all LAV constraints obtained from *τ* by restricting the conclusion to at most *ℓ* atoms. Formally, let *τ* = *A*(**x**) →∃**y** *A*_1_(**x**, **y**) ∧… ∧ *A*_*r*_(**x**, **y**) and let {*j*_1_,…, *j*_*p*_}⊆{1,…*r*} for *p* ≥ 1. Define *τ*[*j*_1_,…, *j*_*p*_]:= $A(\mathbf {x}) \to \exists \mathbf {y}\, A_{j_{1}}(\mathbf {x},\mathbf {y})\land \ldots \land A_{j_{p}}(\mathbf {x},\mathbf {y})$. We define

$$T(\tau,\ell) \text{:=} \{\tau[j_{1},\ldots, j_{p}] \mid \{j_{1},\ldots,j_{p}\}\subseteq \{1,\ldots,r\} \text{ and } p\leq \ell \}. $$

### **Claim C.**

Let (*n*_*i*_)_*i* ≥ 1_ be the strictly increasing sequence of positive integers according to Claim B and let $(\mathcal {M}^{\prime }_{n})_{n\geq 1}$ be the sequence of LAV mappings constructed above. Then, for every *ℓ* ≥ 1, the following properties hold: (i) for every *n* ≥ *n*_*ℓ*_, we have that $\mathcal {M}^{\prime }_{n} \equiv _{\mathsf {CQ}^{\prime }_{\ell }} \mathcal {M}$; (ii) the conclusion of every LAV constraint in $\mathcal {M}^{\prime }_{n_{\ell }}$ is of length at most *ℓ*.

We now make the following claim about the sequence $(\mathcal {M}^{\prime }_{n})_{n\geq 1}$.

### **Claim D.**

For every source instance *I*, there exists an integer *n*_0_ ≥ 1 such that for every *I* ^′^⊆ *I*, we have that $\text {Sol}(I^{\prime },\mathcal {M}^{\prime }_{n_{0}}) = \text {Sol}(I^{\prime },\mathcal {M})$.

Next, we use Claim D to show that $\mathcal {M}$ is closed under unions, i.e., given $(I_{1},J_{1}) \in \mathcal {M}$ and $(I_{2},J_{2}) \in \mathcal {M}$, we must show that $(I,J) \in \mathcal {M}$ with *I* = *I*_1_ ∪ *I*_2_ and *J* = *J*_1_ ∪ *J*_2_. From Claim D, we know that there exists *n*_0_ such that $\text {Sol}(I^{\prime },\mathcal {M}^{\prime }_{n_{0}}) = \text {Sol}(I^{\prime },\mathcal {M})$, for every *I* ^′^⊆ *I*. In particular, *I*_1_, *I*_2_ ⊆ *I*. Hence, for each *i* ∈{1,2}, we have $J_{i} \in \text {Sol}(I_{i}, \mathcal {M}^{\prime }_{n_{0}})$, that is, $(I_{1}, J_{1})\in \mathcal {M}^{\prime }_{n_{0}}$ and $(I_{2}, J_{2})\in \mathcal {M}^{\prime }_{n_{0}}$. Since $\mathcal {M}^{\prime }_{n_{0}}$ is a LAV mapping, it is closed under unions. Hence, $(I,J) \in \mathcal {M}^{\prime }_{n_{0}}$, and, since $\text {Sol}(I,\mathcal {M}^{\prime }_{n_{0}}) = \text {Sol}(I,\mathcal {M})$, we conclude that $J \in \text {Sol}(I,\mathcal {M})$, i.e., $(I,J)\in \mathcal {M}$.

To complete the proof of the lemma, it remains to prove Claims A-D.

### **Claim A.**

The notions of *u* ^′^-limit and uniform limit coincide. Formally, for every sequence $(\mathcal {M}_{n})_{n\geq 1}$ of schema mappings and every schema mapping $\mathcal {M}$, we have that $\mathcal {M}_{n} \stackrel {u}{\longrightarrow } \mathcal {M}$ if and only if $\mathcal {M}_{n} \stackrel {u^{\prime }}{\longrightarrow } \mathcal {M}$. (⇒) Assume $\mathcal {M}_{n} \stackrel {u}{\longrightarrow } \mathcal {M}$. We have to show that also $\mathcal {M}_{n} \stackrel {u^{\prime }}{\longrightarrow } \mathcal {M}$ holds. Consider an arbitrary *ℓ* ≥ 1 and let *r* be the maximal arity of the target schema of $\mathcal {M}$. Any conjunctive query with at most *ℓ* atoms can have at most *m* = *ℓ* ⋅ *r* variables. Hence, the inclusion *C* *Q* ^′^_*ℓ*_ ⊆*C* *Q*_*m*_ holds.

We are assuming $\mathcal {M}_{n} \stackrel {u}{\longrightarrow } \mathcal {M}$. Hence, there exists *n*_0_(*m*) such that for all *n* ≥ *n*_0_(*m*), we have that $\mathcal {M}_{n} \equiv _{\mathsf {CQ}_{m}} \mathcal {M}$. That is, for all *q* ∈*C* *Q*_*m*_ and for all *I*, it holds that $cert(q, I, \mathcal {M}_{n}) = cert(q, I, \mathcal {M})$. Since *C* *Q* ^′^_*ℓ*_ ⊆*C* *Q*_*m*_, we may conclude that for all *q* ∈*C* *Q* ^′^_*ℓ*_ and for all *I*, it holds that $cert(q, I, \mathcal {M}_{n}) = cert(q, I, \mathcal {M})$. Hence, $\mathcal {M}_{n} \stackrel {u^{\prime }}{\longrightarrow } \mathcal {M}$ indeed holds.

(⇐) Assume $\mathcal {M}_{n} \stackrel {u^{\prime }}{\longrightarrow } \mathcal {M}$. We have to show that also $\mathcal {M}_{n} \stackrel {u}{\longrightarrow } \mathcal {M}$ holds. Consider an arbitrary *m* ≥ 1. As above, let *r* be the maximal arity of the target schema of $\mathcal {M}$. Moreover, let *p* be the number of target relation symbols. Any conjunctive query with at most *m* variables can have at most *ℓ* = *p* ⋅ *m* ^*r*^ atoms. Hence, the inclusion *C* *Q*_*m*_ ⊆*C* *Q* ^′^_*ℓ*_ holds.

We are assuming $\mathcal {M}_{n} \stackrel {u^{\prime }}{\longrightarrow } \mathcal {M}$. Hence, there exists *n*_0_(*ℓ*) such that for all *n* ≥ *n*_0_(*ℓ*), we have that $\mathcal {M}_{n} \equiv _{\mathsf {CQ}^{\prime }_{\ell }} \mathcal {M}$. That is, for all $q \in \mathsf {CQ}^{\prime }_{\ell }$ and for all *I*, it holds that $cert(q, I, \mathcal {M}_{n}) = cert(q, I, \mathcal {M})$. Since *C* *Q*_*m*_ ⊆*C* *Q* ^′^_*ℓ*_, we may conclude that for all *q* ∈*C* *Q*_*m*_ and for all *I*, it holds that $cert(q, I, \mathcal {M}_{n}) = cert(q, I, \mathcal {M})$. Hence, $\mathcal {M}_{n} \stackrel {u}{\longrightarrow } \mathcal {M}$ indeed holds.

### **Claim B.**

Assume that $\mathcal {M}_{n} \stackrel {u}{\longrightarrow } \mathcal {M}$. Then, there exists a strictly increasing sequence (*n*_*i*_)_*i* ≥ 1_ of positive integers, such that for every *ℓ* ≥ 1 and for every *n* ≥ *n*_*ℓ*_, we have that $\mathcal {M}_{n} \equiv _{\mathsf {CQ}^{\prime }_{\ell }} \mathcal {M}$. Since $\mathcal {M}_{n} \stackrel {u^{\prime }}{\longrightarrow } \mathcal {M}$, for each *ℓ* ≥ 1 there exists an integer $n^{\prime }_{\ell }$ such that for all $n \geq n^{\prime }_{\ell } $, we have that $\mathcal {M}_{n} \equiv _{\mathsf {CQ}^{\prime }_{\ell }} \mathcal {M}$. We may choose *n*_*ℓ*_ as follows to ensure strict monotonicity: $n_{1} \text {:=}\, n^{\prime }_{1}$

…$n_{\ell } \text {:=}\, \max (n_{\ell -1} + 1, n^{\prime }_{\ell })$Then the sequence (*n*_*i*_)_*i* ≥ 1_ is strictly increasing and for all *ℓ* ≥ 1 and for all *n* ≥ *n*_*ℓ*_, we have that $\mathcal {M}_{n} \equiv _{\mathsf {CQ}^{\prime }_{\ell }} \mathcal {M}$.

### **Claim C.**

Let (*n*_*i*_)_*i* ≥ 1_ be the strictly increasing sequence of positive integers according to Claim B and let $(\mathcal {M}^{\prime }_{n})_{n\geq 1}$ be the sequence of LAV mappings constructed above. Then, for every *ℓ* ≥ 1, the following properties hold: (i) for every *n* ≥ *n*_*ℓ*_, we have that $\mathcal {M}^{\prime }_{n} \equiv _{\mathsf {CQ}^{\prime }_{\ell }} \mathcal {M}$; (ii) the conclusion of every LAV constraint in $\mathcal {M}^{\prime }_{n_{\ell }}$ is of length at most *ℓ*. Consider an arbitrary *ℓ* ≥ 1. By the construction of the sequence $(\mathcal {M}^{\prime }_{n})_{n\geq 1}$, every LAV constraint in $\mathcal {M}^{\prime }_{n}$ has a conclusion of length at most *ℓ*. Hence, property (ii) clearly holds.

To prove property (i), consider an arbitrary *n* ≥ *n*_*ℓ*_. We have to show that $\mathcal {M}^{\prime }_{n} \equiv _{\mathsf {CQ}^{\prime }_{\ell }} \mathcal {M}$, i.e., for arbitrary source instance *I* and arbitrary conjunctive query *q* ∈*C* *Q* ^′^_*ℓ*_, we have to show that $cert(q, I, \mathcal {M}^{\prime }_{n}) = cert(q, I, \mathcal {M})$. By Claim B, we have $\mathcal {M}_{n} \equiv _{\mathsf {CQ}^{\prime }_{\ell }} \mathcal {M}$. Hence, it suffices to show that $cert(q, I, \mathcal {M}^{\prime }_{n}) = cert(q, I, \mathcal {M}_{n})$ holds. We prove the two inclusions separately.

By the construction of $\mathcal {M}^{\prime }_{n}$, we clearly have $chase(I,\mathcal {M}^{\prime }_{n}) \to chase(I,\mathcal {M}_{n})$. From this, it follows immediately that $cert(q,I,\mathcal {M}^{\prime }_{n}) \subseteq cert(q,I,\mathcal {M}_{n})$.

For the reverse inclusion, consider an arbitrary tuple $\mathbf {a} \in cert(q, I,\mathcal {M}_{n})$. Then, there exists a homomorphism $h_{n} \colon q \to chase(I,\mathcal {M}_{n})$ with *h*(**z**) = **a**, where **z** denotes the free variables of *q*. Let $h_{n}(q) = \{A_{1}, {\ldots } A_{k}\} \subseteq chase(I,\mathcal {M}_{n})$ with *k* ≤ *ℓ*. By construction, $\mathcal {M}^{\prime }_{n}$ is obtained by restricting the conclusions of the LAV constraints $\tau \in \mathcal {M}_{n}$ in all possible ways to at most *ℓ* atoms. Hence, since *k* ≤ *ℓ*, we have that also $chase(I,\mathcal {M}^{\prime }_{n})$ contains the set {*A*_1_,…*A*_*k*_} of atoms (up to renaming of labeled nulls). Thus, there exists a homomorphism $h \colon \{A_{1}, {\ldots } A_{k}\} \to chase(I,\mathcal {M}^{\prime }_{n})$ and *h*(*h*_*n*_(⋅)) is a homomorphism $q \to chase(I,\mathcal {M}^{\prime }_{n})$ with *h*(**z**) = **a**. Therefore, $\mathbf {a} \in cert(q, I,\mathcal {M}^{\prime }_{n})$ holds.

Before presenting the proof of Claim D, we need to bring the notion of *fact block size* into the picture; this notion was introduced in [7].

**Fact Blocks**. Let *J* be an instance. The *Gaifman graph of facts* *G*_*J*_ of *J* is the graph whose nodes are the facts of *J* and there is an edge between two facts if they have a null in common. The fact blocks (or f-blocks) of *J* are the sets of nodes of the connected components of *G*_*J*_. The *block size* of an undirected graph *G* is the size of the maximal connected component of *G*_*J*_, where the size of a component is given as the number of nodes. The *fact block size* (f-block size) of an instance *J* is the block size of the Gaifman graph of facts of *J*.

### **Claim D.**

For every source instance *I*, there exists an integer *n*_0_ ≥ 1 such that for every *I* ^′^⊆ *I*, we have that $\text {Sol}(I^{\prime },\mathcal {M}^{\prime }_{n_{0}}) = \text {Sol}(I^{\prime },\mathcal {M})$. Consider an arbitrary *I* ^′^⊆ *I*. Let *J* denote a universal solution for *I* ^′^ w.r.t. $\mathcal {M}$ and let $J^{\prime } = chase(I^{\prime },\mathcal {M}^{\prime }_{n_{0}})$. We set *ℓ* = size(*J*), where size (*J*) denotes the number of atoms in *J*. Moreover, we set *n*_0_ = *n*_*ℓ*_ from the construction of $(\mathcal {M}^{\prime }_{n})_{n\geq 1}$. We claim that *n*_0_ has the desired property. The proof proceeds in three steps, namely, we will show (i) *J* → *J* ^′^, (ii) *J* ^′^→ *J*, and, finally, (iii) $\text {Sol}(I^{\prime },\mathcal {M}^{\prime }_{n_{0}}) = \text {Sol}(I^{\prime },\mathcal {M})$.

- (i) Let **u** = (*u*_1_,…, *u*_*i*_) in *J* denote the labeled nulls in *J* and let **y** = (*y*_1_,…, *y*_*i*_) denote a vector of pairwise distinct variables. Consider the boolean conjunctive query ∃**y** *q*_*J*_ whose atoms are the atoms in *J* where we instantiate the labeled nulls **u** = (*u*_1_,…, *u*_*i*_) with **y** = (*y*_1_,…, *y*_*i*_). Clearly *q*_*J*_ → *J* holds and, therefore, also $cert(\exists \mathbf {y}\, q_{J}, I^{\prime },\mathcal {M}) = true$.

Since $\mathcal {M}^{\prime }_{n_{0}} \equiv _{\mathsf {CQ}^{\prime }_{\ell }} \mathcal {M}$ and ∃**y** *q*_*J*_ ∈*C* *Q* ^′^_*ℓ*_, also $cert(\exists \mathbf {y}\, q_{J}, I^{\prime }, \mathcal {M}^{\prime }_{n_{0}}) = \mathit {true}$ holds. Hence, there exists a homomorphism *h* ^′^: *q*_*J*_ → *J* ^′^, which can be easily transformed into a homomorphism *h*: *J* → *J* ^′^ by setting *h*(*u*_*α*_) = *h* ^′^(*y*_*α*_) for every *α* ∈{1,…, *i*}.

- (ii) For every f-block *F* ^′^ of *J* ^′^, we consider the boolean conjunctive query $\exists \mathbf {z}\, q_{F^{\prime }}$ whose atoms are the atoms in *F* ^′^ and **z** = (*z*_1_,…, *z*_*i*_) instantiates the labeled nulls **v** = (*v*_1_,…, *v*_*i*_) in *F* ^′^ with pairwise distinct variables. Clearly, for every *F* ^′^, we have $q_{F^{\prime }} \to J^{\prime }$ and, therefore, also $cert(\exists \mathbf {z}\, q_{F^{\prime }}, I^{\prime },\mathcal {M}) = true$.

Since all LAV-constraints in $\mathcal {M}^{\prime }_{n_{0}}$ have conclusion size bounded by *ℓ*, the number of atoms in any f-block of *J* ^′^ is bounded by *ℓ*. Hence, for every *F* ^′^, the corresponding conjunctive query $q_{F^{\prime }}$ is in $\mathsf {CQ}^{\prime }_{\ell }$. Since $\mathcal {M}^{\prime }_{n_{0}} \equiv _{\mathsf {CQ}^{\prime }_{\ell }} \mathcal {M}$, we have that $cert(\exists \mathbf {z}\, q_{F^{\prime }}, I^{\prime }, \mathcal {M}) = \mathit {true}$. Hence, for every f-block *F* ^′^ of *J* ^′^, there exists a homomorphism $h_{F^{\prime }} \colon q_{F^{\prime }} \to J$, which can easily be transformed into a homomorphism $g_{F^{\prime }} \colon F^{\prime } \to J^{\prime }$ by setting $g_{F^{\prime }}(v_{\alpha }) = h_{F^{\prime }}(z_{\alpha })$ for every *α* ∈{1,…, *i*}. These homomorphisms from the f-blocks of *J* ^′^ to *J* can be combined to the desired homomorphism $h^{\prime } = \bigcup g_{F^{\prime }}$ with *h* ^′^: *J* ^′^→ *J*.

- (iii) Finally, we show that $\text {Sol}(I^{\prime },\mathcal {M}^{\prime }_{n_{0}}) = \text {Sol}(I^{\prime },\mathcal {M})$ holds.

“ ⊆”: Let $K \in \text {Sol}(I^{\prime },\mathcal {M}^{\prime }_{n_{0}})$. Since *J* ^′^ is a universal solution for *I* ^′^ w.r.t. $\mathcal {M}^{\prime }_{n_{0}}$, there exists a homomorphism *g* ^′^ : *J* ^′^→ *K*. By composing *g* ^′^ with the homomorphism *h* : *J* → *J* ^′^, we obtain a homomorphism from *J* to *K*. By the closure under target homomorphisms, we conclude that $K \in \text {Sol}(I^{\prime },\mathcal {M})$

“ ⊇”: Now let $K \in \text {Sol}(I^{\prime },\mathcal {M})$. Since *J* is a universal solution for *I* ^′^ w.r.t. $\mathcal {M}$, there exists a homomorphism *g* : *J* → *K*. By composing *g* with the homomorphism *h* ^′^ : *J* ^′^→ *J*, we obtain a homomorphism from *J* ^′^ to *K*. Since LAV mapping $\mathcal {M}^{\prime }_{n_{0}}$ is closed under target homomorphisms, we conclude that $K \in \text {Sol}(I^{\prime },\mathcal {M}^{\prime }_{n_{0}})$.

The proof of Lemma 3 is now complete.

□

We now have all the tools needed to present the proof of Theorem 5. Before doing so and for the sake of readability, we reproduce its statement.

Let $(\mathcal {M}_{n})_{n\geq 1}$ be a premise-bounded sequence of GLAV mappings. The following statements are equivalent.

1. $(\mathcal {M}_{n})_{n\geq 1}$ has a GLAV mapping $\mathcal {M}$ as a uniform limit.
2. $(\mathcal {M}_{n})_{n\geq 1}$ has a uniform limit that admits universal solutions.

Moreover, if $(\mathcal {M}_{n})_{n\geq 1}$ is a sequence of LAV mappings, then $(\mathcal {M}_{n})_{n\geq 1}$ has a LAV mapping as a uniform limit if and only $(\mathcal {M}_{n})_{n\geq 1}$ has a uniform limit that admits universal solutions.

### *Proof 14* (Proof of Theorem 5)

The direction (1) ⇒ (2) is obvious. For the direction (2) ⇒ (1), we start with the case when $(\mathcal {M}_{n})_{n\geq 1}$ is a sequence of LAV mappings.

Assume that $\mathcal {M}$ is a uniform limit of a sequence $(\mathcal {M}_{n})_{n\geq 1}$ of LAV mappings and that $\mathcal {M}$ admits universal solutions. Without loss of generality, we may also assume that $\mathcal {M}$ is closed under target homomorphism. Indeed, if we let $\mathcal {M}^{\prime }$ be the schema mapping obtained by closing $\mathcal {M}$ under target homomorphisms, then $\mathcal {M}^{\prime }$ is also a uniform limit of $(\mathcal {M}_{n})_{n\geq 1}$ and it admits universal solutions; this is so because the notion of uniform limit is based on *C* *Q*-equivalence and also conjunctive queries are preserved under homomorphisms. Then the schema mapping $\mathcal {M}$ has the following properties:

1. $\mathcal {M}$ allows for *C* *Q*-rewriting (by Lemma 2);
2. $\mathcal {M}$ admits universal solutions (by hypothesis);
3. $\mathcal {M}$ is closed under target homomorphisms (by hypothesis);
4. $\mathcal {M}$ is closed under unions (by Lemma 3).

Theorem 3.1 in [19] asserts that if a schema mapping admits universal solutions, allows for query rewriting, and is closed under both target homomorphisms and unions, then it is logically equivalent to a LAV mapping. Consequently, we have that $\mathcal {M}$ is logically equivalent to a LAV mapping.For the case when $(\mathcal {M}_{n})_{n\geq 1}$ is a sequence of premise-bounded GLAV mappings (but not necessarily LAV mappings), we apply yet another structural characterization of GLAV mappings from [19], namely, Theorem 3.9, which asserts that if a schema mapping allows for *C* *Q*-rewriting, admits universal solutions, is closed under target homomorphisms, and is n-modular, for some fixed n, then it is logically equivalent to a GLAV mapping.

Let k be the constant bounding the length of premises in $(\mathcal {M}_{n})_{n\geq 1}$. We proceed exactly as in the proof of Lemma 3 and construct a sequence $(\mathcal {M}^{\prime }_{n})_{n\geq 1}$, in which the premises of tgds are the same as in tgds in $(\mathcal {M}_n)_{n\geq 1}$, hence each tgd in $(\mathcal {M}^{\prime }_{n})_{n\geq 1}$ has at most k atoms in its premise. We proceed exactly as in the proof of Lemma 3 to establish the following analog of Claim D.*Claim D *(*in the proof of Lemma 3*) *For every source instance* *I*, *there exists an integer* *n*_0_ ≥ 1 *such that for* *every* *I* ^′^⊆ *I* *, we* *have that* $\text {Sol}(I^{\prime },\mathcal {M}^{\prime }_{n_{0}}) = \text {Sol}(I^{\prime },\mathcal {M})$.

Now, since each tgd in every element of $(\mathcal {M}^{\prime }_{n})_{n\geq 1}$ has at most k atoms in its premise, it follows that there is a positive integer *N*_*k*_ so that each mapping $\mathcal {M}^{\prime }_{n}$ in $(\mathcal {M}^{\prime }_{n})_{n\geq 1}$ is *N*_*k*_-modular. It is easy to see that *N*_*k*_ ≤ *k* ⋅ *r* holds where r is the maximum relation arity in the source schema.

We now prove that $\mathcal {M}$ is *N*_*k*_-modular. Assume that *J* is not a solution for I w.r.t. to $\mathcal {M}$. Take an integer *n*_0_ as in Claim D and consider $\mathcal {M}^{\prime }_{n_{0}}$. It follows that *J* is not a solution for I w.r.t. $\mathcal {M}^{\prime }_{n_{0}}$. Since $\mathcal {M}^{\prime }_{n_{0}}$ is *N*_*k*_-modular, there is a subinstance *I* ^′^ of I such that *J* is not a solution for *I* ^′^ w.r.t. $\mathcal {M}^{\prime }_{n_{0}}$ and |*d* *o* *m*(*I* ^′^)|≤ *N*_*k*_. Again by Claim D, we have that *J* is not a solution for *I* ^′^ w.r.t. $\mathcal {M}$, hence M is *N*_*k*_-modular.

Thus, $\mathcal {M}$ has the following properties: it admits *C* *Q*-rewriting (since it is the uniform limit of GLAV mappings that admit *C* *Q*-rewriting), it admits universal solutions, is closed under target homomorphisms (if it is not, we take its closure before we begin the construction), and, as just shown, it is *N*_*k*_-modular. Consequently, by Theorem 3.9 in [19], we have that $\mathcal {M}$ is logically equivalent to a GLAV schema mapping, which completes the proof. □

We conclude this section with a conjecture concerning uniform limits of arbitrary sequences of GLAV mappings.

### **Conjecture 1**

*The following statements are equivalent for a sequence* $(\mathcal {M}_{n})_{n\geq 1}$ *of* *GLAV mappings.*

1. $(\mathcal {M}_{n})_{n\geq 1}$ *has an SO tgd as a uniform limit.*
2. $(\mathcal {M}_{n})_{n\geq 1}$ *has a uniform limit that admits universal solutions.*

It is not hard to show that the preceding conjecture is implied by a conjecture in [2] to the effect that the language of *plain* SO-tgds^2^ can be characterized by the following three properties: allowing for *C* *Q*-rewriting, admitting universal solutions, and closure under target homomorphisms.

## Metric Space Completion and Generalized Schema Mappings

Let **T** be a schema containing a binary relation symbol. By Proposition 2, the metric space $(\mathcal {P}(\text {Inst}(\mathbf {T})),dist)$ is not complete, i.e., there are Cauchy sequences of elements of $\mathcal {P}(\text {Inst}(\mathbf {T}))$ that have no limit in $\mathcal {P}(\text {Inst}(\mathbf {T}))$. Let $(\mathcal {P}(\text {Inst}(\mathbf {T}))^{*},dist^{*})$ be the completion of $(\mathcal {P}(\text {Inst}(\mathbf {T})),dist)$. As described in Section 2, the elements of $\mathcal {P}(\text {Inst}(\mathbf {T}))^{*}$ are the equivalence classes of Cauchy sequences of elements of $\mathcal {P}(\text {Inst}(\mathbf {T}))$, where two Cauchy sequences $\mathcal {I}_{1},\mathcal {I}_{2},\ldots $ and $\mathcal {J}_{1},\mathcal {J}_{2},\ldots $ are equivalent if $\lim \limits _{n\to \infty } dist(\mathcal {I}_{n},\mathcal {J}_{n}) = 0$. Clearly, this is a rather abstract description of $\mathcal {P}(\text {Inst}(\mathbf {T}))^{*}$. In this section we show that, in many cases, the elements of $\mathcal {P}(\text {Inst}(\mathbf {T}))^{*}$ can be represented by suitably constructed infinite **T**-instances. In turn, this result and basic results about complete metric spaces imply that the (pointwise or uniform) limits of a Cauchy sequence of schema mappings can be represented by a *generalized* schema mapping, that is, a schema mapping in which infinite solutions are allowed. We also establish a tight connection between these results and the representation of structural limits in the monograph by Nešetřil and Ossona de Mendez [15].

### Representing Limits of Cauchy Sequences in the Metric Completion

Let **T** be a schema. Recall that, by definition, a **T**-instance is a finite set of facts. In what follows, we will also consider infinite **T**-instances, where, by definition, an *infinite* **T**-instance is an infinite set *I* of facts *R*_*i*_(*t*_1_,…, *t*_*m*_). The term **T**-instance will continue to denote a finite **T**-instance, but, at times and for emphasis or disambiguation, we will also use the term finite **T**-instance, especially in contexts in which infinite **T**-instances are also considered. According to Definitions 2 and 3, the notion of the distance between two sets of finite instances has been defined using the notion of *C* *Q*_*n*_-equivalence, where two sets $\mathcal {J}$ and $\mathcal {J}^{\prime }$ of finite **T**-instances are *C* *Q*_*n*_-equivalent, denoted $\mathcal {J} \equiv _{\mathsf {CQ}_{n}} \mathcal {J}^{\prime }$, if it holds that $cert(q,\mathcal {J}) = cert(q,{\mathcal {J}^{\prime }})$, for all *q* ∈*C* *Q*_*n*_. The notion of *C* *Q*_*n*_-equivalence naturally extends to arbitrary (i.e., finite or infinite) **T**-instances. Hence, also the notions of similarity and distance, both of which were defined via *C* *Q*_*n*_-equivalence, immediately carry over to sets of arbitrary **T**-instances. Furthermore, the set of sets of arbitrary **T**-instances forms a pseudometric space, in which we can speak about Cauchy sequences and limits.

#### **Definition 8**

Let **T** be a schema.

- Let $\mathcal {X}$ and $\mathcal {Y}$ be two sets of finite **T**-instances. We say that $\mathcal {Y}$ is an *isomorphic copy of* $\mathcal {X}$ *with nulls named apart* if
  1. For every member *J* of $\mathcal {X}$, there is a member *J* ^′^ of $\mathcal {Y}$ that is an isomorphic copy of *J* via an isomorphism that renames nulls.
  2. Every member *J* ^′^ of $\mathcal {Y}$ is an isomorphic copy of some member *J* of $\mathcal {X}$ via an isomorphism that renames nulls.
  3. No two members of $\mathcal {Y}$ have nulls in common.
- If $\mathcal {Y}$ is a set of finite **T** instances, then $\bigcup {\mathcal {Y}}$ denotes the union of all members of $\mathcal {Y}$ (where each member of $\mathcal {Y}$ is viewed as a set of facts).
- If $\mathcal {X}$ is a set of finite **T**-instances, then $\bigoplus {\mathcal {X}}$ denotes the set consisting of the unions of isomorphic copies of $\mathcal {X}$ with nulls named apart, i.e.,
  $$\bigoplus {\mathcal{X}} = \left\{ \bigcup {\mathcal{Y}}\mid \mathcal{Y}\text{ is an isomorphic copy of }\mathcal{X}\text{ with nulls named apart}\right\}.$$

Several remarks are in order now.

- Let $\mathcal {Y}$ be a set of finite **T**-instances. Clearly, if $\mathcal {Y}$ is finite, then $\bigcup {\mathcal {Y}}$ is a finite **T**-instance, while if $\mathcal {Y}$ is infinite, then $\bigcup {\mathcal {Y}}$ is an infinite **T**-instance. Note also that if $\mathcal {X}$ is a set of finite **T**-instances such that at least one instance in $\mathcal {X}$ contains nulls, then $\bigoplus {\mathcal {X}}$ is infinite (even if $\mathcal {X}$ is a finite set).
- According to Definition 4, if *J* is a **T**-instance whose active domain contains nulls only, then *v*(*J*) is the set of all **T**-instances that are isomorphic copies of *J* via an isomorphism that renames nulls. This notation makes sense also for infinite **T**-instances *J* whose active domains contain nulls only. With this in mind, observe that if $\mathcal {X}$ is a finite set of **T**-instances and if $\mathcal {Y}$ is an isomorphic copy of $\mathcal {X}$ with nulls named apart, then
  $$\bigoplus {\mathcal{X}}= v(\bigcup {\mathcal{Y}}).$$
- As a concrete example, if ${\mathcal {K}} = \{K_{n} \mid n\geq 1\}$, where *K*_*n*_ is a clique of size *n* in which every node is a null, then the members of $\bigoplus {\mathcal {K}}$ are precisely the disjoint unions of cliques of all finite sizes in which every node is a null.

#### **Definition 9**

Let q be a conjunctive query over the schema **T** with k free variables, *k* ≥ 0, and let **a** be a k-tuple of constants (if *k* = 0, then **a** = (), i.e., **a** is the empty tuple).

We write *q*(**a**) to denote the **T**-instance *J* obtained from q and **a** by (i) substituting the free variables of q by the respective elements of **a**; (ii) replacing the existential variables of q by fresh distinct labeled nulls; and (iii) treating the resulting body atoms of q as facts of the **T**-instance *J*.

Note that if *q* is a boolean query (in which case **a** = ()), then *q*(()) is the *canonical database* of *q*, i.e., the **T**-instance whose active domain is the set of variables of *q* viewed as distinct nulls and whose facts are the atoms of *q*. Conversely, every **T**-instance *J* whose active domain consists entirely of nulls is the canonical database of a boolean conjunctive query.

Before stating the main result of this section, we need to introduce one more concept. Let $\mathcal {J}$ be a set of finite or infinite **T**-instances. We say that $\mathcal {J}$ is *closed under isomorphisms that rename nulls* if for every (finite or infinite) **T**-instance *J* in $\mathcal {J}$ and for every (finite or infinite) **T**-instance *J* ^′^ that is an isomorphic copy of *J* via an isomorphism that renames nulls, we have that *J* ^′^ is also in $\mathcal {J}$. Note that if $\mathcal {X}$ is a set of finite **T**-instances, then $\bigoplus {\mathcal {X}}$ is closed under isomorphisms that rename nulls. Moreover, if ${\mathcal {M}}$ is a schema mapping between **S** and **T**, then, for every source instance *I*, the set $\text {Sol}(I,{\mathcal {M}})$ of the solutions of *I* w.r.t. $\mathcal {M}$ is closed under isomorphisms that rename nulls (see Definition 1).

#### **Theorem 6**

*Let* $(\mathcal {J}_{n})_{n\geq 1}$ *be a Cauchy sequence of elements of* $\mathcal {P}(\text {Inst}(\mathbf {T}))$ *such that each* $\mathcal {J}_{n}$ *is closed under isomorphisms that rename nulls. Then the limit of the sequence* $(\mathcal {J}_{n})_{n\geq 1}$ *is the set* $\bigoplus \mathcal {J}^{*}$ *,* *where*

$$\begin{array}{@{}rcl@{}} &&\mathcal{J}^{*} = \{ q(\mathbf{a}) \mid q \in \mathsf{CQ}\text{ \textit{and there is an integer }p\textit{ such that}}\\ &&{\kern142pt}\mathbf{a} \in \text{ cert}(q,\mathcal{J}_i),\text{ \textit{for every} }i\geq p \}. \end{array} $$

#### *Proof 15*

We have to show that, for every *m* ≥ 1, there is some *n*_0_ such that for every *q* ∈*C* *Q*_*m*_ and every *n* ≥ *n*_0_, we have that $cert(q,\mathcal {J}_{n})= cert(q,\bigoplus \mathcal {J}^{*})$. This will be done in two steps, as follows.

- Step 1: We will show that, for every *m* ≥ 1, there is some *n*_1_ such that $cert(q,\mathcal {J}_{n}) \subseteq cert(q,\bigoplus \mathcal {J}^{*})$, for every *q* ∈*C* *Q*_*m*_ and every *n* ≥ *n*_1_.
- Step 2: We will show that, for every *m* ≥ 1, there is some *n*_2_ such that $ cert(q,\bigoplus \mathcal {J}^{*})\subseteq cert(q,\mathcal {J}_{n})$, for every *q* ∈*C* *Q*_*m*_ and every *n* ≥ *n*_2_.

Then, given *m* ≥ 1, we can take *n*_0_ = max{*n*_1_, *n*_2_}.

We start by pointing out that for every *n* ≥ 1 and every *q* ∈*C* *Q*, the certain answers $cert(q,\mathcal {J}_{n})$ consist entirely of null-free tuples. This follows from the assumption that $\mathcal {J}_{n}$ is closed under isomorphisms that rename nulls (the proof is essentially the same as the proof of Proposition 1 in Section 2). Moreover, for every *q* ∈*C* *Q*, the certain answers $cert(q,\bigoplus \mathcal {J}^{*})$ also consist entirely of null-free tuples. This is so because $\bigoplus \mathcal {J}^{*}$ contains isomorphic copies of $\mathcal {J}^{*}$ having no nulls in common (e.g., if *v*_1_,…, *v*_*n*_,… is a list of all nulls, then $\bigoplus \mathcal {J}^{*}$ contains an isomorphic copy of $\mathcal {J}^{*}$ in which all nulls have even index and an isomorphic copy of $\mathcal {J}^{*}$ in which all nulls have odd index). Thus, we only need to focus on tuples of constants as possible certain answers.

To prove Step 1, since the sequence $(\mathcal {J}_{n})_{n\geq 1}$ is Cauchy, for every *m* ≥ 1, there is some *n*_1_ such that if *s* ≥ *n*_1_ and *t* ≥ *n*_1_, then $\mathcal {J}_{s} \equiv _{\mathsf {CQ}_{m}} \mathcal {J}_{t}$. We now claim that $cert(q,\mathcal {J}_{n}) \subseteq cert(q,\bigoplus \mathcal {J}^{*})$, for every *q* ∈*C* *Q*_*m*_ and every *n* ≥ *n*_1_. Indeed, assume that *q* ∈*C* *Q*_*m*_ and let **a** be a (possibly empty) tuple of constants in $cert(q,\mathcal {J}_{n})$, where *n* ≥ *n*_1_. It follows that $\mathbf {a} \in cert(q,\mathcal {J}_{j})$, for every *j* ≥ *n*_1_, hence the finite **T**-instance *q*(**a**) is in the set $\mathcal {J}^{*}$. Consequently, $\mathbf {a} \in q(\bigcup {\mathcal {Y}})$, for every isomorphic copy $\mathcal {Y}$ of $\mathcal {J}^{*}$ with nulls named apart, which implies that $\mathbf {a} \in cert(q,\bigoplus \mathcal {J}^{*})$.

To prove Step 2, we will first show that the set D of constants occurring in $ \mathcal {J}^{*}$ is finite (note that D is also the set of constants occurring in $\bigoplus \mathcal {J}^{*}$). As a stepping stone, we will show the finiteness of a set *D* ^′^ that is defined next.

A single-atom conjunctive query is a query of the form ∃**y** *R*(**x**, **y**), where R is a relation symbol in the schema **T**. Let *D* ^′^ be the set of all constants b for which there is a single-atom query q and an index p, such that b occurs in $cert(q,\mathcal {J}_{i})$, for all *i* ≥ *p*. We claim that the set *D* ^′^ is finite. To see this, observe first that every single-atom query has at most r variables, where r is the maximum arity of the relation symbols in **T**. Since the sequence $(\mathcal {J}_{n})_{n\geq 1}$ is Cauchy, there exists an integer *p*_*r*_ such that $\mathcal {J}_{i} \equiv _{\mathsf {CQ}_{r}} \mathcal {J}_{p_{r}}$, for all *i* ≥ *p*_*r*_. This implies that the certain answers to single-atom conjunctive queries become fixed in $(\mathcal {J}_{n})_{n\geq 1}$ starting from the index *p*_*r*_, which depends only on the schema **T**. By definition, the certain answers hold in every instance in $\mathcal {J}_{p_{r}}$. Since $\mathcal {J}_{p_{r}}$ consists entirely of finite instances, the set *D* ^′^ must be finite as well.

To complete the proof of the finiteness of D, we will show that *D* ⊆ *D* ^′^. Let **a** be a tuple of constants for which there is a conjunctive query q and an index p, such that $\mathbf {a} \in cert(q,\mathcal {J}_{i})$, for all *i* ≥ *p*. Let s be the number of atoms of q and consider the single-atom queries $q^{\prime }_{1}(\mathbf {y}_{i}), \dots , q^{\prime }_{s}(\mathbf {y}_{s})$ that cover *q* in the following sense: for every *j* with 1 ≤ *j* ≤ *s*, the atom of $q^{\prime }_{j}$ is the j-th atom of q, and **y**_*j*_ contains exactly the free variables of q that occur in this atom. Let **a**_*j*_ be the tuple of elements from **a** assigned to the variables **y**_*j*_. Clearly, every element of **a** is an element of some **a**_*j*_, 1 ≤ *j* ≤ *s*. Observe that $\mathbf {a}_{j} \in cert(q, \mathcal {J}_{i})$ implies that $\mathbf {a}_{j} \in cert(q^{\prime }_{j}, \mathcal {J}_{i})$, hence we have that $\mathbf {a}_{j} \in cert(q^{\prime }_{j},\mathcal {J}_{i})$, for every *i* ≥ *p*. Thus, each element of **a**_*j*_, 1 ≤ *j* ≤ *s*, is an element of *D* ^′^. This shows that *D* ⊆ *D* ^′^ holds, hence D is a finite set.

We now return to the proof of Step 2. We will show that for every *m* ≥ 1, there is some *n*_2_ such that $ cert(q,\bigoplus \mathcal {J}^{*})\subseteq cert(q,\mathcal {J}_{n})$, for every *q* ∈*C* *Q*_*m*_ and every *n* ≥ *n*_2_. Assume that *q* ∈*C* *Q*_*m*_ and let **a** be a tuple of constants such that $\mathbf {a} \in cert(q,\bigoplus \mathcal {J}^{*})$. Then, for every instance $J \in \bigoplus \mathcal {J}^{*}$, we have that **a** ∈ *q*(*J*), hence there is a homomorphism h from the variables of q to the active domain of*J* such that the tuple of the free variables of q is mapped to **a** and the atoms of q are mapped to facts of*J*. Let s be the number of atoms of q and let *f*_1_,…, *f*_*s*_ be the facts of*J* that are the images of the atoms of q under the homomorphism h. Up to renaming nulls, each fact *f*_*j*_ is a fact of some finite **T**-instance of the form *q*_*j*_(**b**_*j*_), where *q*_*j*_ is a conjunctive query and **b**_*j*_ is a tuple of constants such that $\mathbf {b}_{j} \in cert(q_{j},\mathcal {J}_{i})$, for all sufficiently large i. Let *n*_*q*(**a**)_ be an index such that for every *i* ≥ *n*_*q*(**a**)_, we have that $\mathbf {b}_{j} \in cert(q_{j},\mathcal {J}_{i})$ holds, for 1 ≤ *j* ≤ *s*. Furthermore, let *n*_2_ be the maximum such index *n*_*q*(**a**)_, for all q in *C* *Q*_*m*_ and for all tuples **a** in D. Such an index exists (i.e., it is a finite number) because both the set *C* *Q*_*m*_ and the set of tuples of elements D of length at most m is finite.

Observe that *n*_2_ has been chosen so that for every tuple **a** and for every *q* ∈*C* *Q*_*m*_ with a homomorphism h mapping *q*(**a**) to some instance in $\bigoplus \mathcal {J}^{*}$ (and thus to every instance in $\bigoplus \mathcal {J}^{*}$, by renaming the nulls in the co-domain of h accordingly), every fact *f*_*j*_ in *h*(*q*(**a**)) can be mapped further to every instance $\mathcal {J}_{n}$, *n* ≥ *n*_2_, via a homomorphism *h*_*i*_ defined on the entire f-block of *f*_*j*_. (Recall that, by the definition of $\bigoplus \mathcal {J}^{*}$, each fact *f*_*j*_ instantiates an atom of some conjunctive query *q*_*j*_ whose certain answers persist in the sequence $(\mathcal {J}_{n})_{n\geq 1}$; the bodies of these queries are mapped into instances of $\bigoplus \mathcal {J}^{*}$ after renaming apart the nulls in them, thus ensuring that no two distinct queries end up in the same f -block of an instance of $\bigoplus \mathcal {J}^{*}$).

The union of two homomorphisms *h*_1_, *h*_2_ defined on two distinct f-blocks *B*_1_, *B*_2_ is unambiguously defined, and it is a homomorphism on the instance *B*_1_ ∪ *B*_2_, since homomorphisms are the identity on constants and f-blocks do not share nulls. Thus, for an instance $J \in \bigoplus \mathcal {J}^{*}$ and for the image {*f*_1_,…, *f*_*s*_} of *q*(**a**) under some homomorphism h, we also have a homomorphism from *q*(**a**) to *J*_*n*_, *n* ≥ *n*_2_, obtained by composing h with a union *h*_1_ ∪⋯ ∪ *h*_*s*_ of homomorphisms from the f-blocks of the atoms *f*_1_,…, *f*_*s*_ to *J*_*n*_. It follows that $\mathbf {a} \in cert(q,\mathcal {J}_{n})$, for every *n* ≥ *n*_2_. This establishes the inclusion $cert(q,\mathcal {J}^{*}) \subseteq cert(q,\mathcal {J}_{n})$, for *n* ≥ *n*_2_, and completes the proof of the theorem. □

Recall the sequence (*v*(*K*_*n*_))_*n* ≥ 1_ in Proposition 2, where *K*_*n*_ is the clique of size *n* whose vertices are pairwise distinct labeled nulls. By Proposition 2, this sequence is Cauchy, but has no limit in $\mathcal {P}(\text {Inst}(\mathbf {T}))$. Theorem 6 tells us how to find the limit in the complete metric space via the conjunctive queries with non-empty certain answers over all but finitely many members of the sequence. Since the instances *K*_*n*_, *n* ≥ 1, have active domains consisting entirely of nulls, Lemma 1 tells us that we only need to consider boolean conjunctive queries and, moreover, it suffices to evaluate them on each *K*_*n*_. These queries can only use the edge relation *E*, thus they can be considered as graphs - with the variables representing the vertices. If a query contains a self-loop (i.e., an atom of the form *E*(*z*, *z*) for some variable *z*), then the query evaluates to false over every *K*_*n*_. On the other hand, if a query contains no self-loop, then it evaluates to true over all but finitely many instances *K*_*n*_. Indeed, let *q* be a conjunctive query without self-loop and suppose that *q* contains *m* variables. It is easy to verify that *q* evaluates to true over all instances *K*_*n*_ with *n* ≥ *m*. Hence, by Theorem 6, the limit of (*v*(*K*_*n*_))_*n* ≥ 1_ is $\bigoplus {\mathcal {G}}$, where $\mathcal {G}$ is a set of graphs with the following properties: (i) every member of $\mathcal {G}$ is a graph with no self-loops and with labelled nulls as vertices; (ii) every graph with no self-loops is isomorphic to a graph in $\mathcal {G}$. Clearly, $\bigoplus {\mathcal {K}}$ is also the limit of (*v*(*K*_*n*_))_*n* ≥ 1_, where ${\mathcal {K}}$ is a set of graphs with the following properties: (i) every member of $\mathcal {K}$ is a clique with labelled nulls as vertices; (ii) every clique is isomorphic to a graph in ${\mathcal {K}}$. Thus, the limit of (*v*(*K*_*n*_))_*n* ≥ 1_ is the set consisting of all disjoint unions of cliques of all finite sizes in which every node is a null. At any rate, it is clear that infinite instances have to be used to represent the limit of (*v*(*K*_*n*_))_*n* ≥ 1_.

Next, we extend our results about limits of Cauchy *sequences of instances* to limits of Cauchy *sequences of mappings*. To this end, we first recall two basic results about complete metric spaces.

#### **Proposition 8**

*Let* (*Y*, *d*)*be a complete* *metric space and let* (*f*_*n*_)_*n* ≥ 1_ *be a sequence of functions from a set* X *to* Y.

- *If* (*f*_*n*_)_*n* ≥ 1_ *is a pointwise Cauchy sequence, then* (*f*_*n*_)_*n* ≥ 1_ *has a pointwise limit* *f* : *X* → *Y* *,* *where* $f(x) = \lim \limits _{n\to \infty } f_{n}(x)$ *,* *for every* *x* ∈ *X*.
- *If* (*f*_*n*_)_*n* ≥ 1_ *is a uniformly Cauchy sequence, then* (*f*_*n*_)_*n* ≥ 1_ *has a uniform limit. Moreover, the pointwise limit* *f* : *X* → *Y* *of* (*f*_*n*_)_*n* ≥ 1_ *is also the uniform limit of* (*f*_*n*_)_*n* ≥ 1_.

The proof of the first part of Proposition 8 is immediate from the definitions; the proof of the second part can be found in any standard book on metric spaces (see, e.g., Proposition 3.6.6 in [18]). In fact, the argument is essentially the same as the one given in the proof of Part 2 of Theorem 4. Note that the second part of Proposition 8 is known as the *Cauchy criterion*.

We are now ready to obtain concrete representations of the (pointwise or uniform) limits of Cauchy sequences of schema mappings.

#### **Definition 10**

Let **S**, **T** be two schemas. A generalized schema mapping is a set $\mathcal {M}$ of pairs (*I*, *J*) such that I is a finite **S**-instance,*J* is a finite or infinite **T**-instance, and $\mathcal {M}$ has the following closure property: if $(I,J)\in \mathcal {M}$ and if *J* ^′^ is an isomorphic copy of*J* via an isomorphism that renames nulls, then $(I,J^{\prime }) \in \mathcal {M}$.

#### **Corollary 3**

*Let* $(\mathcal {M}_{n})_{n\geq 1}$ *be a sequence of schema mappings. Consider the generalized schema* *mapping*

$$\mathcal{M} = \left\{(I,J) \mid J \in \bigoplus \{ q(\mathbf{a}) \mid q \in \mathsf{CQ} \wedge \exists p\ \forall i \geq p\ (\mathbf{a} \in cert(q,I,\mathcal{M}_{i})) \} \right\}. $$

- If $(\mathcal {M}_{n})_{n\geq 1}$is a pointwise Cauchy sequence, then the schema mapping$\mathcal {M}$is the pointwise limit of $(\mathcal {M}_{n})_{n\geq 1}$.
- If $(\mathcal {M}_{n})_{n\geq 1}$is a uniformly Cauchy sequence, then the schema mapping$\mathcal {M}$is the uniform limit of $(\mathcal {M}_{n})_{n\geq 1}$.

#### *Proof 16*

The first part follows from Theorem 6 and the definitions. The second part follows from the first part and Proposition 8. □

Finally, we consider (pointwise or uniformly) Cauchy sequences of schema mappings admitting universal solutions and obtain a different representation of their limits.

#### **Corollary 4**

*Let* $(\mathcal {M}_{n})_{n\geq 1}$ *be a pointwise Cauchy sequence of schema mappings over a source schema* **S** *and a target* *schema* **T** *,* *each admitting universal solutions.*

1. *For every* *I* ∈Inst(**S**)*,* *the sequence* $(\text {UnivSol}(I, \mathcal {M}_{n}))_{n\geq 1}$ *is Cauchy, and hence it has a limit* $\lim \limits _{n\to \infty }(\text {UnivSol}(I, \mathcal {M}_{n}))$ *in the complete metric space* $(\mathcal {P}(\text {Inst}(\mathbf {T}))^{*},dist^{*})$.
2. *The generalized schema* *mapping*
  $$\mathcal{M}^{*} = \{(I, J) \mid I \in \text{Inst}(\mathbf{S}), J \in \lim\limits_{n\to \infty}(\text{UnivSol}(I, \mathcal{M}_{n}))\} $$
  is a pointwise limitof $(\mathcal {M}_{n})_{n\geq 1}$. Moreover, if$(\mathcal {M}_{n})_{n\geq 1}$is a uniformly Cauchysequence, then $\mathcal {M}^{*}$is its uniform limit.

### Connections with Representations of Structural Limits

In their recent monograph [15], Nešetřil and Ossona de Mendez considered a notion of distance between instances, as well as sequences of instances and limits of such sequences. In what follows, we describe the main differences between their setting and ours.

- The first main difference is that they did not distinguish two classes of domain elements (namely, constants and nulls), as we did here. As a result, in the definition of homomorphism in [15], no special treatment of constants is needed, while, in our setting, constants must always be mapped to themselves. Their notion of homomorphism coincides with ours on instances whose active domains consist of labeled nulls only. Note that this is exactly the scenario we had in Example 1 and Proposition 2, which are both inspired by results in [15].
- The second main difference is that the notion of distance in [15] is between a pair of two instances, while our notion of distance is between a pair of two *sets* of instances. This, of course, raises the question of how the two notions compare if, in our setting, both sets are singletons. We will address this question soon.
- The third main difference is that, when cast in terms of the certain answers of conjunctive queries, the notion of distance in [15] involves boolean conjunctive queries only, while ours involves all conjunctive queries (boolean and non-boolean ones).

In what follows, we recall the definition of the similarity measure and the metric from [15] and briefly sketch the approach that Nešetřil and Ossona de Mendez took in representing limits of Cauchy sequences of instances via infinite instances.

Let **T** be a schema and let *J* and *J* ^′^ be two **T**-instances. By a slight abuse of notation, we write *J* → *J* ^′^ to denote the existence of a homomorphism from *J* to *J* ^′^ in the sense of Nešetřil and Ossona de Mendez (i.e., not distinguishing two types of domain elements). As mentioned before, if the active domains of *J* and *J* ^′^ contain nulls only, then this notion of homomorphism coincides with the one considered in the context of schema mappings and data exchange (which is the one we used here).

#### **Definition 11**

[Left distance in [15]] Let **T** be a schema and let *J*, *J* ^′^ be two **T**-instances.

- The similarity *s* *i* *m*_*h*_(*J*, *J* ^′^) between *J* and *J* ^′^ is the size of the active domain of a smallest instance B such that one of the following two conditions holds: (a) *B* → *J* and $B \nrightarrow J^{\prime }$; (b) $B \nrightarrow J$ and *B* → *J* ^′^. If no such finite instance B exists, we let *s* *i* *m*_*h*_(*J*, *J* ^′^) = *∞*.
- The distance *d* *i* *s* *t*_*h*_(*J*, *J* ^′^) between *J* and *J* ^′^ is the quantity $dist_{h}(J,J^{\prime }) = 2^{-sim_{h}(J,J^{\prime })}$.

Nešetřil and Ossona de Mendez call this distance the “left distance”, because it is defined in terms of homomorphisms *from* other structures. This is to distinguish the notion from the “right distance” which is defined in terms of homomorphisms *to* other structures. For our purposes here, only the left distance is relevant. Because of the basic connection between homomorphisms and boolean conjunctive queries, it is easy to see that if *J* and *J* ^′^ are **T**-instances, then the following statements are equivalent.

- *s* *i* *m*_*h*_(*J*, *J* ^′^) = *m*.
- *m* is the largest number such that *J* and *J* ^′^ satisfy the same boolean conjunctive queries with at most *m* − 1 variables.

How do the notions of *s* *i* *m*_*h*_ of similarity and *d* *i* *s* *t*_*h*_ of distance compare with our notions *sim* of similarity and *dist* of distance? Clearly, this comparison is meaningful only when, in our setting, we consider singletons of instances and, moreover, the active domains of these instances contain nulls only. Recall that, according to the notation introduced in Definition 4, if *J* is a **T**-instance whose active domain contains nulls only, then *v*(*J*) is the set of all **T**-instances that are isomorphic copies of *J* via an isomorphism that renames nulls. The next observation is a direct consequence of Definitions 3 and 11, Lemma 1, and the preceding remarks.

#### **Proposition 9**

*Let* **T** *be a schema* *and let* *J* *and* *J* ^′^ *be two* **T** *-instances* *whose active domains contain nulls only. Then the following statements are* *true.*

- *s* *i* *m*(*v*(*J*), *v*(*J* ^′^)) = *s* *i* *m*_*h*_(*J*, *J* ^′^) − 1.
- *d* *i* *s* *t*(*v*(*J*), *v*(*J* ^′^)) = 2 ⋅ *d* *i* *s* *t*_*h*_(*I*, *I* ^′^).

In what follows, we will write NInst(**T**) to denote the set of all **T**-instances whose active domain consists entirely of nulls. The pair (NInst(**T**), *d* *i* *s* *t*_*h*_) is a pseudometric space, so a metric space can be obtained from it by passing to the equivalence classes [*J*] of target instances *J*, where [*J*] consists of all target instances that are homomorphically equivalent to *J*. As we did for the distance *dist* and the pseudometric space $(\mathcal {P}(\text {Inst}(\mathbf {T})),dist)$, we will identify each equivalence class with one of its members.

Cauchy sequences and limits arising from *d* *i* *s* *t*_*h*_ are called *left Cauchy sequences* and *left limits* in [15]. Proposition 9 implies that if (*J*_*n*_)_*n* ≥ 1_ is a sequence of elements of NInst(**T**), then (*J*_*n*_)_*n* ≥ 1_ is Cauchy with respect to the distance *d* *i* *s* *t*_*h*_ if and only if the sequence (*v*(*J*_*n*_))_*n* ≥ 1_ is Cauchy with respect to the distance *dist*. If (*J*_*n*_)_*n* ≥ 1_ is a sequence of elements of NInst(**T**), then we will write $\lim \limits ^{h}_{n\to \infty } J_{n}$ for the limit of the sequence (*J*_*n*_)_*n* ≥ 1_ in the metric completion $(\text {NInst}(\textbf {T})^{*},dist^{*}_{h})$ of the space (NInst(**T**), *d* *i* *s* *t*_*h*_). Nešetřil and Ossona de Mendez obtained representations of the left limits of Cauchy sequences of instances by an approach that is based on the *homomorphism preorder* on instances and on *ideals* of partial orders.

The existence of homomorphisms between structures gives rise to the preorder ≤_*h*_, where *L*≤_*h*_ *J* if *L* → *J*. By passing to the equivalence classes [*J*] of instances *J* in NInst(**T**) modulo homomorphic equivalence, the preorder ≤_*h*_ becomes a partial order (also denoted by ≤_*h*_), where [*L*] ≤_*h*_[*J*] means that there is a homomorphism from some member of [*L*] to some member of [*J*]; this is the same as asserting that, for every pair (*L* ^′^, *J* ^′^) with *L* ^′^∈ [*L*] and *J* ^′^∈ [*J*], there is a homomorphism from *L* ^′^ to *J* ^′^. As before, we will not distinguish between equivalence classes and their members. The partial order ≤_*h*_ extends to a partial order $\leq _{h}^{*}$ on the metric completion $(\text {NInst}(\textbf {T})^{*},dist^{*}_{h})$ of (NInst(**T**), *d* *i* *s* *t*_*h*_) in the following way.

- If (*J*_*n*_)_*n* ≥ 1_ and (*L*_*n*_)_*n* ≥ 1_ are two Cauchy sequences from NInst(**T**), then $\lim \limits _{n\to \infty }^{h} L_{n} \leq _{h}^{*} \lim \limits _{n\to \infty }^{h} J_{n}$ if for every *m*, there is a positive integer *p* such that for every *i* ≥ *p*, we have that $\min \{|B|: B\to L_{i}~\text {and}~B \nrightarrow J_{i}\} \geq m$.
- As a special case, it is easy to see that if *L* is an element of NInst(**T**) and (*J*_*n*_)_*n* ≥ 1_ is a Cauchy sequence from NInst(**T**), then $L \leq _{h}^{*} \lim \limits _{n\to \infty }^{h} J_{n}$ holds if and only if there is a positive integer *p* such that for every *i* ≥ *p*, we have that *L* → *J*_*i*_ (this is the special case of $\lim \limits _{n\to \infty }^{h} L_{n} \leq _{h}^{*} \lim \limits _{n\to \infty }^{h} J_{n}$ in which *L*_*n*_ = *L*, for all *n*).

Let (*X*,≤) be a (finite or infinite) partially ordered set.

- A *downset* is a subset *F* of *X* with the property that for all *x* ∈ *F* and *y* ≤ *x*, also *y* ∈ *F* holds.
- An *ideal* is a downset *F* with the additional property that for all *x* and *y* in *F*, there exists *z* in *F* such that both *x* ≤ *z* and *y* ≤ *z* hold.

In [15], it is shown that there is a correspondence between left limits of Cauchy sequences from NInst(**T**) and ideals in the partial order (NInst(**T**),≤_*h*_). Before presenting this correspondence, we need to introduce a piece of notation.

If ${\mathcal {X}}$ is a set of **T**-instances, then the *disjoint union* $\biguplus {\mathcal {X}}$ is the set $\bigcup {\mathcal {Y}}$, where ${\mathcal {Y}}$ is an isomorphic copy of ${\mathcal {X}}$ with nulls named apart. In other words, $\biguplus {\mathcal {X}}$ is the union of copies of all elements of $\mathcal {X}$ (one copy of each element of $\mathcal {X}$) so that no two members in the union have nulls in common. Clearly, $\biguplus {\mathcal {X}}$ is unique up to isomorphisms that rename nulls.

Let (*J*_*n*_)_*n* ≥ 1_ be a Cauchy sequence from NInst(**T**) and let $\lim \limits _{n\to \infty }^{h}J_{n}$ be the left limit of (*J*_*n*_)_*n* ≥ 1_ in the metric completion $(\text {NInst}(\textbf {T})^{*},dist^{*}_{h})$ of the space (NInst(**T**), *d* *i* *s* *t*_*h*_). Consider the set

$$\{L \in \text{NInst}(\textbf{T})\mid L \leq_{h}^{*} \lim\limits_{n\to \infty}^{h}J_{n}\}. $$

It is easy to see that this set is an ideal of (NInst(**T**),≤_*h*_). Indeed, it is a downset because homomorphisms compose. Moreover, if *L*_1_ and *L*_2_ are in $\{L\in \text {NInst}(\textbf {T})\mid L \leq _{h}^{*} \lim \limits _{n\to \infty }^{h}J_{n}\}$, then so is the disjoint union $\biguplus \{L_{1},L_{2}\}$ of *L*_1_ and *L*_2_; moreover, $L_{i}\leq _{h} \biguplus \{L_{1},L_{2}\}$, for *i* = 1,2. The following is a consequence of Lemma 9.6 and Corollary 9.3 in [15].

#### **Proposition 10** (15)

*The following statements are true for the complete metric space* $(\text {NInst}(\textbf {T})^{*},dist^{*}_{h})$ *and the partial* *order* $(\text {NInst}(\textbf {T})^{*},\leq _{h}^{*})$.

- *There is a bijection* $\mathcal {F}$ *between* NInst(**T**)^∗^ *and the set of ideals of* (NInst(**T**),≤_*h*_)*given by* ${\mathcal {F}}(\lim \limits _{n\to \infty }^{h}J_{n}) = \{L \in \text {NInst}(\textbf {T})\mid L \leq _{h}^{*} \lim \limits _{n\to \infty }^{h}J_{n}\}.$
- *If* $\lim \limits _{n\to \infty }^{h}J_{n}$ *is the left limit* *of a Cauchy sequence* (*J*_*n*_)_*n* ≥ 1_ *from* NInst(**T**)*,* *then* $\lim \limits _{n\to \infty }^{h}J_{n}$ *can be represented as the disjoint union of the associated ideal* ${\mathcal {F}}(\lim \limits _{n\to \infty }J_{n})$ *,* *namely,*
  $$\lim\limits_{n\to \infty}^{h}J_{n} = \biguplus \{L \in \text{NInst}(\textbf{T})\mid L \leq_{h}^{*} \lim\limits_{n\to \infty}^{h}J_{n}\}. $$

We now have all the conceptual and technical apparatus needed to establish a tight connection between the representations of limits given in Theorem 6 and the representation of limits given in Proposition 10.

Let {*J*_*n*_}_*n* ≥ 1_ be a Cauchy sequence (w.r.t. the distance function *d* *i* *s* *t*_*h*_) such that each *J*_*n*_ is a member of NInst(**T**), i.e., each *J*_*n*_ is a **T**-instance whose active domain consists entirely of nulls. Let $\lim \limits _{n\to \infty }^{h}J_{n}$ be its left-limit in the metric completion of (NInst(**T**), *d* *i* *s* *t*_*h*_). As discussed earlier, the sequence {*v*(*J*_*n*_)}_*n* ≥ 1_ is Cauchy (w.r.t. the distance function *dist*), so it has a limit $\lim \limits _{n\to \infty }v(J_{n})$ in the metric completion of $(\mathcal {P}(\text {Inst}(\mathbf {T})),dist)$. The following proposition establishes the close relationship between these two limits.

#### **Proposition 11**

*Let* {*J*_*n*_}_*n* ≥ 1_ *be a Cauchy sequence (w.r.t. the distance function* *d* *i* *s* *t*_*h*_ *) such that* *each* *J*_*n*_ *is a* *member of* NInst(**T**).*Then*

$$\lim\limits_{n\to \infty}v(J_{n}) = v(\lim\limits_{n\to \infty}^{h}J_{n}). $$

#### *Proof 17*

Theorem 6 tells us that

$$\lim\limits_{n\to \infty}v(J_{n})= \bigoplus \{ q(\mathbf{a}) \mid q \in \mathsf{CQ} \wedge \exists p \ \forall i\geq p \ (\mathbf{a} \in cert(q, \{v(J_{i})\}))\}. $$

Since the active domains of the elements of *v*(*J*_*n*_) consist entirely of nulls and are pairwise disjoint, we have that only boolean conjunctive queries *q* contribute to this expression. Moreover, by Lemma 1, the condition **a** ∈ *c* *e* *r* *t*(*q*,{*v*(*J*_*i*_)}) means that *c* *e* *r* *t*(*q*,{*J*_*i*_}) = *t* *r* *u* *e* or, equivalently, that *J*_*i*_⊧*q*. As mentioned earlier, every boolean conjunctive query *q* can be identified with its canonical database *D*_*q*_. Moreover, *J*_*i*_⊧*q* if and only if *D*_*q*_ → *J*_*i*_. Thus, the preceding equation becomes

$$\lim\limits_{n\to \infty}v(J_{n})= \bigoplus \{L\in \text{NInst}(\textbf{T})\mid \exists p \ \forall i\geq p \ (L\to J_{i})\}.$$

As explained earlier, the condition ∃*p* ∀*i* ≥ *p* (*L* → *J*_*i*_) is equivalent to the condition $L \leq _{h} \lim \limits _{n\to \infty }^{h}J_{n}$, hence the preceding equation becomes

$$\lim\limits_{n\to \infty}v(J_{n})=\bigoplus \{L\in \text{NInst}(\textbf{T})\mid L \leq_{h} \lim\limits_{n\to \infty}^{h}J_{n}\}.$$

This last equation and the second remark after Definition 8 imply that indeed

$$\lim\limits_{n\to \infty}v(J_{n}) = v(\lim\limits_{n\to \infty}^{h}J_{n}).$$

□

## Concluding Remarks

**Fig. 1:**
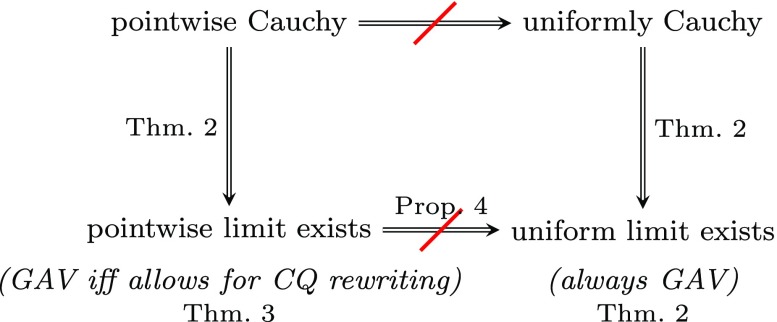
Overall picture for GAV schema mappings

**Fig. 2:**
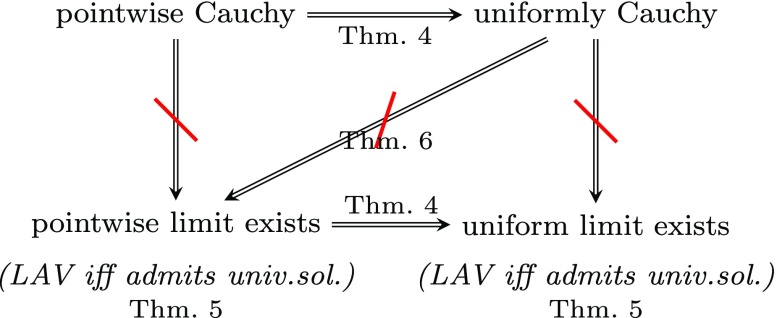
Overall picture for LAV schema mappings

In this paper, we have embarked on a systematic study of the limiting behavior of sequences of schema mappings using concepts and tools from metric spaces. For the important special cases of GAV and LAV mappings, our main results are summarized in Figs. 1 and 2.

In words, we have shown that, for GAV mappings, a pointwise Cauchy sequence need not be uniformly Cauchy; moreover, the existence of a pointwise limit does not imply the existence of a uniform limit. This cannot happen for LAV mappings. On the other side, a uniformly Cauchy sequence of LAV mappings need not even have a pointwise limit, which cannot happen for GAV mappings. We have also shown that structural properties of schema mappings can be used to characterize when the limit of a pointwise Cauchy sequence of GAV (or of LAV) mappings is equivalent to a GAV (or to a LAV) mapping. Finally, we have shown that infinite target instances and generalized mappings (i.e., schema mappings where target instances may be infinite) can be used to represent limits of Cauchy sequences of sets of target instances and limits of Cauchy sequences of arbitrary schema mappings.

We believe that the work reported here has laid the foundation for several interesting lines of subsequent investigations. We have seen that our results about sequences of LAV mappings extend in a natural way to sequences of premise-bounded GLAV mappings; an analogous extension of our results about sequences of GAV mappings to sequences of *conclusion-bounded* GLAV mappings is left for future work. We have also seen that there are sequences of LAV mappings for which no SO tgd is a uniform limit. Are there structural properties that characterize when a sequence of GLAV mappings has an SO tgd as a pointwise limit? In this vein, we have offered Conjecture 1. A related interesting open problem is whether schema mappings with target constraints are powerful enough to express pointwise limits or uniform limits of sequences of arbitrary GLAV schema mappings. We have some preliminary evidence that this is plausible, but much more work remains to be done.

We believe that the work reported in this paper provides a new perspective on the study of schema mappings by examining them from a dynamic viewpoint. As stated earlier, our original motivation came from schema-mapping optimization and, in particular, from the idea that “complex” schema mappings can be “approximated” by “simpler” ones. It remains to be seen whether the work reported here will lead to applications to schema-mapping optimization. We believe, however, that the study of the limiting behavior of schema mappings via metric spaces is interesting in its own right.

We also note there are several areas in theoretical computer science where the study of limiting behavior of objects has produced results that were significant in their own right and also had fruitful consequences. For example, starting with the work of Fagin [4], there has been an extensive investigation of the asymptotic probabilities of logical properties and of 0-1 laws for various logics of interest in computer science. More recently, there has been a study of *profinite words*, which has found applications to automata theory and to the satisfiability problem for variants of monadic second-order logic (see, e.g., [17, 20]). Note that the profinite words form the completion of a metric space on words in which the distance is based on the size of the largest deterministic finite automaton needed to separate two words. Finally, the connection between graph limits in the monograph [15] by Nešetřil and Ossona de Mendez and the completion of the metric space $(\mathcal {P}(\text {Inst}(\mathbf {T})),d)$, which was mentioned in the previous section, may merit further exploration. It should also be pointed out that, motivated by the study of large-scale networks, there has been an extensive body of work on a notion of graph limits arising from converging sequences of *homomorphism densities*; a detailed account of this work is given in the monograph [13] by Lovász. In addition, Nešetřil and Ossona de Mendez [16] developed a general framework for limits of graphs and relational structures; in that framework, different fragments of first-order logic are used to define different notions of limits arising from converging sequences of the frequencies that first-order formulas in the fragment at hand are satisfied by an assignment (homomorphism densities correspond to the fragment consisting of all quantifier-free conjunctive queries). Homomorphisms, metric completions, and representations of limits of finite structures play a central role in [13, 16].

## Footnotes

1 Allowing for *C* *Q*-rewriting means that the certain answers of every conjunctive query over the target schema is definable by a union of conjunctive queries over the source schema - see [19].

2 A *plain* SO tgd is an SO tgd that contains no nested terms and no equalities. Every SO tgd is known to be *C* *Q*-equivalent to a plain one [2].
